# *Moringa oleifera* (drumstick tree)—nutraceutical, cosmetological and medicinal importance: a review

**DOI:** 10.3389/fphar.2024.1288382

**Published:** 2024-02-02

**Authors:** Marta Klimek-Szczykutowicz, Katarzyna Gaweł-Bęben, Angelika Rutka, Eliza Blicharska, Małgorzata Tatarczak-Michalewska, Katarzyna Kulik-Siarek, Wirginia Kukula-Koch, Magdalena Anna Malinowska, Agnieszka Szopa

**Affiliations:** ^1^ Department of Pharmaceutical Sciences, Collegium Medicum, Jan Kochanowski University, Kielce, Poland; ^2^ Department of Cosmetology, University of Information Technology and Management in Rzeszów, Rzeszów, Poland; ^3^ Chair and Department of Pharmaceutical Botany, Faculty of Pharmacy, Medical College, Jagiellonian University, Cracow, Poland; ^4^ Department of Pathobiochemistry and Interdisciplinary Applications of Ion Chromatography, Biomedical Sciences, Medical University of Lublin, Lublin, Poland; ^5^ Department of Pharmacognosy with Medicinal Plants Garden, Medical University of Lublin, Lublin, Poland; ^6^ Department of Organic Chemistry and Technology, Faculty of Chemical Engineering and Technology, Cracow University of Technology, Cracow, Poland

**Keywords:** *Moringa oleifera*, drumstick tree, chemical composition, nutritional properties, pharmacological activities, cosmetology applications, agronomic importance

## Abstract

*Moringa oleifera* Lam. (Moringaceae) is a species of tree with an increasing utility, occurring naturally mainly in Pakistan and northern India. *M. oleifera* is currently cultivated in Africa, South America, Asia and the Middle East. The usage of its leaves, seed oil, bark, fruits, flowers and roots has positive opinions of FDA (American Food and Drug Administration), EFSA (European Food Safety Authority) and CosIng (Cosmetic Ingredients database). The chemical composition of *M. oleifera* is dominated by: proteins (consisting mainly of amino acids such as arginine or serine), fatty acids (omega-3 and omega-6), vitamins (vitamin A, B and C and tocopherols), mineral salts (including several bioelements, such as calcium, magnesium, sodium, and potassium), valuable polyphenolic compounds from the group of phenolic acids (e.g., gallic acid, ferulic acid) and flavonoids (e.g., myricetin, rutoside, and kaempferol). The raw materials show antioxidant, hepatoprotective, anti-inflammatory and antimicrobial properties. Dietary supplements and alimentary products containing *M. oleifera* are recommended as health-promoting and “novel food” preparations. The main purpose of this work was a review of the latest scientific literature on *M. oleifera*, with particular emphasis on the studies focusing on its chemical composition, biological activity and safety. Moreover, the review tends to discuss the results of biotechnological studies using this material and the agronomical significance.

## 1 Introduction

*Moringa oleifera* Lam. (drumstick tree) is a tropical tree from the family Moringaceae. *M. oleifera* occurs naturally mainly in Pakistan and northwest India. Currently, this species is widely cultivated, primarily in India and several countries in Africa, South America, Asia and the Middle East (Nelson and Rau, 2011). Thanks to its health-promoting and nutritional properties, *M. oleifera* is called a “miracle tree”, “the tree of long life” or “the horseradish tree” because its roots are bearing a similar taste to horseradish. Due to the content of valuable bioactive compounds, *M. oleifera* combines nutritional and pharmaceutical values. Nutraceuticals are used to supplement the diet with substances of natural origin, which are present in food in limited amounts, and show properties conducive to maintaining health and maintaining the proper functioning of the body (Himanshu et al., 2018).

The leaves of *M. oleifera* (*Moringae folium*) are the main raw material used in the food and cosmetics industries. The drumstick tree belongs to the group of highly useful species that easy to grow and occur naturally in developing countries. This is a species which has been grown in many countries especially in Asia and Africa for centuries, and widely used by local communities for many purposes including as a food and local medication. The growing demand for this species has contributed to its intensive exploitation. Currently, not only the leaves are used as a raw material, but also its fruit, flower, bark, seed and root (Vergara-Jimenez et al., 2017).

*M. oleifera* provides products of high nutritional value, which is why the species is also referred to as “super foods”, “novel foods” and/or “healthy foods” (Joshi and Mehta, 2010). The species of interest contains, among others: proteins (amino acids), fatty acids (omega-3 and omega-6), vitamins (tocopherols), mineral salts (calcium, magnesium)*,* phenolic acids (like gallic acid, ferulic acid) and flavonoids (myricetin) (Bennett et al., 2003; Anwar and Rashid, 2007; Sultana and Anwar, 2008; Sreelatha and Padma, 2009; Joshi and Mehta, 2010; Zhang, 2011; El-Massary et al., 2013; Ijarotimi et al., 2013; Wu et al., 2020). The latest scientific research confirms the health-promoting properties of *M. oleifera* known from the traditional medicine of India, China and Africa. Among the proved pharmacological properties of the tree its antiviral (Florence, 2014), antiinflammatory (Ezeamuzie et al., 1996; Maurya and Singh, 2014), hepatoprotective (Toppo et al., 2015; Sun et al., 2019) and antidepressant (Kaur et al., 2015) properties are confirmed.

*M. oleifera* is listed in the Ayurvedic pharmacopoeia of India only (Government of India Ministry of Ayush, 2016), as it does not have any WHO (World Health Organization) (WHO, 2023) nor EMA—HMPC (European Medicines Agency Committee on Herbal Medicinal Products) (EMA, 2023) monograph. This species, as a food plant, is mentioned in the documents of the FDA (American Food and Drug Administration) (FDA, 2023) and EFSA (European Food Safety Authority) (EFSA, 2023). In the food industry *M. oleifera* is used in the production of cakes, cereal gruels, bread, biscuits, dairy products, soups, salads and teas (Stevens et al., 2013; Hekmat et al., 2015; Karim et al., 2015). It is used not only in cuisine, food industry and dietetics, but also due to its valuable properties, is of high importance to herbal medicines and cosmetology. The tree can also be used to produce biofuels, whereas the plant’s residues are applied as an ecological fertilizer (in line with the “zero waste” trend) (Joshi and Mehta, 2010; Ali et al., 2013; El-Massary et al., 2013).

The review summarizes the information on the botanical, ecological and chemical characteristics of *M. oleifera*. The tradition of use and the current place of the species in agronomy, medicine and cosmetology are presented. Also, scientific research on its biological activities was reviewed and the safety of *M. oleifera* was discussed. In addition, biotechnological studies conducted using *in vitro* cultures of the drumstick tree are also described.

## 2 Botanical and ecological characteristics

*M. oleifera* is a tree with low environmental requirements, characterized by a rapid growth (Vergara-Jimenez et al., 2017). The tree reaches an average height of about 5–10 m. Some sources mention its height as of 1–12 m. Moringa leaves are composed of an odd number of leaflets 15–35 cm long. The leaflets are hairy, obovate, 4–24 mm in size. The flowers of *M. oleifera* are fragrant, 1–3.3 cm in diameter. They have white petals, 1–1.4 cm in size, and larger petals, which are also white or often colored red, and greenish at the base, measuring 1.2–3.3 cm. The inflorescence is a wide, spreading panicle. The fruit is a pod with 3 ribbed lobes. The pods are 18–32 cm long and 0.9–2.2 cm wide and contain about 26 seeds. The seeds are spherical, three-winged, 6–7 cm in size and about 1 cm in diameter. One tree produces between 3,000 and 9,000 seeds per season, and some sources give numbers as high as 15,000 to 25,000 (Vergara-Jimenez et al., 2017).

The *Moringa* species is drought tolerant, which makes it successful in growing in subtropical and tropical countries and in dry to humid tropical or subtropical climates at altitudes from 0 to 1,400 m above sea level (or up to 2,000 m above sea level depending on the source). The annual irrigation requirement for *M. oleifera* is low and varies between 760–2,500 mm of precipitation. The temperature suitable for the development of *M. oleifera* is 18°C–28°C (Nouman et al., 2014).

The natural (primary) sites of origin of *M. oleifera* are located in South Asia. It grows at the foot of the Himalayas in the area from north-eastern Pakistan to the northern state of West Bengal in India and north-eastern Bangladesh. *M. oleifera* is common in countries such as Nepal, Afghanistan, Sri Lanka, Bangladesh, India, Cambodia, Philippines, Hawaii (Nouman et al., 2014). *M. oleifera* has many names in different languages (Ramachandran et al., 1980; Fuglie, 1999; Parrotta, 2009; Paikra et al., 2017; Sujatha and Patel, 2017; World Flora Online, 2022; Trees for Life, 2023). A summary of *M. oleifera* nomenclature is provided in Supplementary Materials. *M. oleifera* is currently intensively cultivated on almost all continents in the equatorial area of Asia, Central America, South America, from Mexico to Peru, in Paraguay, Brazil and South Florida. The production of *M. oleifera* is of particular importance in tropical developing countries as it is an efficient source of food for both humans and animals (Mouchili et al., 2019).

## 3 Crops and cultivars

The traditional cultivation of *M. oleifera*, by seed, takes place in Pakistan, northwest India and Sudan. Vegetative propagation is used in Indonesia and West Africa and finally commercial cultivation of *M. oleifera* is conducted primarily in the other regions of India and Africa as well as Asia, South America and the Middle East (Nouman et al., 2014).

*M. oleifera* seeds germinate within 2 weeks of sowing (up to 2 cm). In nurseries, they are repotted 3–6 weeks after germination, when they reach a height of about 30 cm. Depending on the *M. oleifera* ecotype, the number of germinating seeds per kilogram is from 3 to 9 thousand. Ideal conditions for seed storage are around 3°C with a humidity of 5%–8% (Mouchili et al., 2019).

Cutting is another method used to propagate *M. oleifera*, by planting hard branches from adult trees (4–16 cm in diameter, and 1–2 m long) during the rainy season. After burying a third of the branches in the soil, they easily develop roots which grow to a considerable size within a few months and the plant develops (Nouman et al., 2014). In the literature, there are differences in the benefits of seedlings and seeds. According to Ramachandran et al. (Ramachandran et al., 1980) plants grown from seeds produce lower quality fruit, while Animashaun et al. (Animashaun et al., 2016) state that growing from seed brings benefits for plant stability and access to water by developing longer roots than the trees grown from the cuttings. *M. oleifera* is classified as a very fast growing tree. During the first 3–4 years, its annual growth is 1–2 m per year, and after a few years it reaches 12 m. Topping or pruning is standard practice to support side branches and to shape for easy harvesting (Nouman et al., 2014).

There are several types of *M. oleifera* leaf production. The most intensive production at which fertilization and irrigation is necessary is a dense spacing of 10 by 10 cm–20 by 20 cm, then the leaf harvest period is reached after 35–45 days. The second type is planting at a spacing of about 50 by 100 cm, this is referred to as semi-intensive production. In this type, only irrigation and fertilizer use is suggested, and the period between harvests is between 50 and 60 days. The last type is the largest spaced plant spacing in the agro-forestry system. Irrigation and fertilization of this type of crop is not necessary. The distances between the rows are quite large, between 2 and 4 m, and harvesting is carried out approximately every 60 days. There is a variability in yield and harvest frequency between the described types of *M. oleifera* crops (Oliveira et al., 1999), the time of year and the rainy season play a major role in both. More research is needed to assess the optimal spacing and harvest time of *M. oleifera* (Nouman et al., 2014).

*M. oleifera* leaves can be harvested mechanically and manually. They consist in cutting shoots at a height of 0.5–1 m above the ground. You can also pluck the leaves directly from the tree, this is a less effective technique as it leads to slower regrowth (Ahmed et al., 2016).

The tree bears fruit in the first year after its planting. Between 6 months and 3 years after planting, *M. oleifera* gives a small yield of fruit. After 3 years of planting, the tree can produce from 600 to 1,600 fruits per year. Depending on the cultivar, the plants show differences, and the pod production time varies from 6 months to over a year. Within 6 months new pods develop from pruning the branches (Ahmed et al., 2016).

Seed production is based on the collection of fruit (pods). Each pod contains approximately 26 seeds. After about 3 months become dry and should be harvested as soon as possible. One tree produces about 3,000 to 9,000 seeds (some sources even say 15,000 to 25,000) (Ahmed et al., 2016).

Different ecotypes of *M. oleifera* are cultivated in India, producing fruit with different characteristics; *Chemmurungai* (with distinctive red-tipped pods), *Jaffna* and *Chavakacheri* (with soft fruits), *Palmurungai* and *Punmurungai* (with bitter-tasting fruits), *Kadumurungai* (with small fruits), *Kodikalmurungai* (with short fruits). Ecotypes *Puna Murungai*, *Palmurungai*, *Kodikkal Murungai* are characterized by a high yield of pods. Unfortunately, despite the high variability in breeding databases, there is a lack of precise information on the genetic variability of the plant (Mouchili et al., 2019).

Low soil requirements and resistance to drought are of particular importance in the cultivation of *M. oleifera.* The plant can be grown in diverse soils, except those that are waterlogged. Slightly alkaline clay and sandy loam soils are considered the best media for this species due to their good drainage. For clay soils and high altitudes, it is necessary to use chemical (urea and nitrates) and organic (cattle, poultry, rabbit manure) fertilizers. Such intensification of production takes place, for example, in Western Cameroon (Mouchili et al., 2019).

## 4 Industrial scale production

Currently, the main producer of *M. oleifera* is India where the fruit is produced at the quantity of 1.1–1.3 million tons per year. The cultivation area of *M. oleifera* in India covers about 380 km^2^. The interest in plant breeding is related to its usefulness, ease, speed of growth and fertility, which is important in the countries most exposed to malnutrition (Gandji et al., 2018). According to the Trade Promotion Council of India (TPC) reports (Trade Promotion Council of India, 2021) *M. oleifera* is becoming more and more popular. The main countries importing leaves and seeds of *M. oleifera* are: United States of America, China, Canada, South Korea, Germany and other European countries. In 2018, the value of the global market for *M. oleifera* products was amounted for 5.5 billion USD, and by 2025 it is planned to exceed 10 billion USD. India is the dominant force in the *M. oleifera* export market, sharing about 80% of the total production volume in the world (Mouchili et al., 2019). The Indian states of Andhra Pradesh, Tamil Nadu and Odisha are of particular importance, as their export growth rate increases by about 26%–28% annually (Mouchili et al., 2019; Trade Promotion Council of India, 2021).

Due to its high nutrient content*, M. oleifera* is described in many sources as an alternative food in developing countries and is therefore widely cultivated there. Nigeria and Cameroon, both of which are considered developing countries, produce significant amounts of raw materials from *M. oleifera*. Trade of *M. oleifera* in Cameroon is twice as profitable as of other leafy vegetables (Gandji et al., 2018). In Nigeria, organic fertilizers derived from *M. oleifera* seeds are used. In addition, *M. oleifera* serves as a vegetable eaten by local people and animals (Ojo et al., 2016).

*M. oleifera* is also grown in Florida, Cuba, Jamaica and commercially in Hawaii and the volcanic island of O’ahu, the fruit production in these areas can be year-round. Fruit varieties of *M. oleifera* such as *Chavakacheri*, *Jaffana* and *Chemmuungai* are particularly popular there (Lynch, 2014).

## 5 Phytochemical characteristic

*M. oleifera* is a species rich in valuable phytocompounds. The most important groups of active compounds in the composition of *M. oleifera* are: carotenoids, tocopherols, flavonoids, phenolic acids, folates, polyunsaturated fatty acids, mineral salts, vitamins (Table 1). Dried leaves are the richest sources of polyphenols in the plant. The polyphenolic content depends however on the environmental conditions, harvesting season, the genetics of the plant, the stage of leaves maturity at the time of harvesting and the applied methods of drying and extraction. The main polyphenols of *M. oleifera* are flavonoids, phenolic acids and catechins (Table 1) (Bennett et al., 2003; Richter et al., 2003; Yang et al., 2008; Moyo et al., 2011).

**TABLE 1:** Phytochemical profile of *M. oleifera* raw materials.

Group of compounds	Compounds	Raw material	References
Phenolic acids	chlorogenic acid, ellagic acid, ferulic acid, gallic acid, caffeic acid, *o-*coumaric acid, syringic acid	leaves	Yang et al. (2008), Moyo et al. (2011)
	chlorogenic acid, ellagic acid, ferulic acid, gallic acid, caffeic acid, syringic acid	pods	Yang et al. (2008)
	chlorogenic acid, ellagic acid, ferulic acid, gallic acid, caffeic acid, syringic acid	seeds	Yang et al. (2008)
Flavonoids	isorhamnetin, kaempferol, quercetin, myricetin, rutin	leaves	Bennett et al. (2003), Richter et al. (2003), Yang et al. (2008), Moyo et al. (2011)
	kaempferol, quercetin, rutin	pods	Yang et al. (2008)
	kaempferol, quercetin, rutin	seeds	Yang et al. (2008)
Catechins	epicatechin, catechin, pirocatechin	leaves	Yang et al. (2008), Moyo et al. (2011), Florence (2014), El-Harairy et al. (2016)
	catechin	pods	Yang et al. (2008)
	catechin	seeds	Yang et al. (2008)
Glucosinolates	4-*O*-(α-L-rhamnopyranosyloxy)-benzyl glucosinolate (glucomoringin)	leaves	Bennett et al. (2003), Ahmed et al. (2016)
	benzylglucosinolate, 4-*O*-(α-L-rhamnopyranosyloxy)-benzyl glucosinolate (glucomoringin)	roots	Bennett et al. (2003)
	4-*O*-(α-L-rhamnopyranosyloxy)-benzyl glucosinolate (glucomoringin)	bark	Bennett et al. (2003)
	4-*O*-(α-L-rhamnopyranosyloxy)-benzyl glucosinolate (glucomoringin)	seeds	Bennett et al. (2003)
Isothiocyanates	4-[4′-*O*-acetylalpha-L-rhamnosyloxy)benzyl] isothiocyanate	leaves	Bennett et al. (2003)
	4 (α-L-rhamnosyloxy) isothiocyanate	seeds	Eilert et al. (1981)
Alkaloids	4′-hydroxyphenylethanamide-α-L-rhamnopyranoside (marumoside A), marmumoside B, pyrrolemarumin, 4″-O-α-L-rhamnopyranoside, methyl 4-(α-L-rhamnopyranosyloxy)-benzylcarbamate, 4-(α- L-rhamnopyranosyloxy)phenylacetonitrile (niazirin), N,α-L-rhamnopyranosylvincosidamide	leaves	Ahmed et al. (2016)
Phenols	pyrogallol	leaves	Yang et al. (2008), Moyo et al. (2011), Florence (2014), El-Harairy et al. (2016)
Vitamins	vitamin A, vitamin B_1_, vitamin B_2_, vitamin B_3_, vitamin C, vitamin E, β-carotene, α-tocopherol	leaves	Joshi and Mehta (2010), Moyo et al. (2011), Zhang (2011), Saini et al. (2014), Ahmed et al. (2016)
	vitamin C	flowers	Vergara-Jimenez et al. (2017)
	vitamin C	pods	Vergara-Jimenez et al. (2017)
	α-tocopherol, γ-tocopherol, δ-tocopherol	seeds	Ijarotimi et al. (2013)
Minerals	zinc, phosphorus, magnesium, manganese, copper, potassium, selenium, sulfur, sodium, calcium, iron	leaves	Sreelatha and Padma (2009)
	magnesium, potassium, calcium, iron	roots	Sreelatha and Padma (2009)
	magnesium, potassium, calcium, iron	bark	Sreelatha and Padma (2009)
	zinc, phosphorus, magnesium, manganese, copper, potassium, selenium, sulphur, sodium, calcium, iron	pods	Sreelatha and Padma (2009)
	zinc, phosphorus, magnesium, potassium, calcium, iron	seeds	Anwar and Rashid (2007)
Fatty acids	arachidic acid, behanic acid, caprylic acid, heicosanoic acid, lauric acid, ligoceric acid, α-linolenic acid, γ-linolenic acid, linolenic acid, margaric acid, myristic acid, oleic acid, palmitylic acid, palmitic acid, stearic acid, tricosanoic acid, vaccenic acid	leaves	Dai et al. (2020)
	arachidic acid, behenic acid, eicosenoic acid, erucic acid, α-linolenic acid, oleic acid, palmitic acid, palmitoleic acid, stearic acid	seeds	Ijarotimi et al. (2013), Athikomkulchai et al. (2021)
Steroles	$\Delta^{5}$ -avenasterol, $\Delta^{7}$ - avenasterol, clerosterol, 28-isoavenasterol, campestanol, $\Delta^{7}$ -campestanol, campesterol, 24-methylene-cholesterol, β-sitosterol, stigmastanol, $\Delta^{7.14}$ -stigmastanol, stigmasterol	seeds	Ijarotimi et al. (2013)
Amino acids	alanine, arginine, cysteine, phenylalanine, glycine, histidine, hydroxyproline, isoleucine, aspartic acid, glutamic acid, leucine, lysine, methionine, proline, serine, threonine, tretinoin, tryptophan, tyrosine, valine	leaves	Yang et al. (2008), Moyo et al. (2011), Dai et al. (2020)
	alanine, arginine, cysteine, phenylalanine, glycine, histidine, isoleucine, aspartic acid, glutamic acid, leucine, lysine, methionine, proline, serine, threonine, tryptophan, tyrosine, valine	pods	Yang et al. (2008)
	alanine, arginine, cysteine, phenylalanine, glycine, hydroxyproline, histidine, isoleucine, aspartic acid, glutamic acid, leucine, lysine, methionine, proline, serine, threonine, tretinoin, tryptophan, tyrosine, valine	seeds	Yang et al. (2008), Moyo et al. (2011)
Carotenoids	13*-cis*-lutein*,* 15-*cis-*β-carotene, *trans*-lutein, *trans*-luteoxin, *trans*- zeaxanthin	leaves	Joshi and Mehta (2010), Moyo et al. (2011), Zhang (2011), Saini et al. (2014)

**FIGURE 1:**
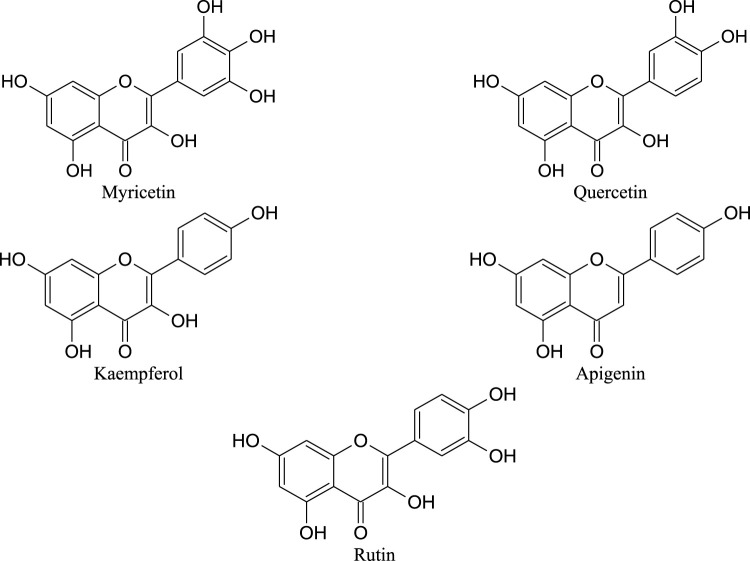
Chemical structures of main flavonoids found in *M. oleifera* leaves.

Flavonoids are present mainly in the leaves of *M. oleifera*. Their estimated values depend on the process to which the leaves were subjected. The main flavonoids found in *M. oleifera* leaves are myricetin, rutin, apigenin, quercetin and kaempferol (Figure 1; Table 2) (Bennett et al., 2003; Richter et al., 2003; Yang et al., 2008; Moyo et al., 2011).

**TABLE 2:** The content of flavonoids in different organs of *M. oleifera* plant.

Metabolites	Raw material	Content (mg/100 g)	References
Total flavonoids	Freeze-dried leaves (Nikaragua)	4.43	Dhakad et al. (2019)
	Freeze-dried leaves (India)	2.10	
	Freeze-dried leaves (Niger)	3.81	
Kaempferol	Fresh leaves	1.80	Yang et al. (2008)
	Dried leaves	0.74	
		40	Richter et al. (2003)
	Degreased pods	22.23	Yang et al. (2008)
	Raw seeds	74.13	
Quercetin	Fresh leaves	28.56	
	Dried leaves	27.14	
		281	Richter et al. (2003)
		3,529	Bennett et al. (2003)
	Degreased pods	42.36	Yang et al. (2008)
	Raw seeds	16.48	
Rutin	Fresh leaves	97.68	
	Dried leaves	89.69	
	Freeze-dried leaves	1,674	Moyo et al. (2011)
	Degreased pods	18.54	Yang et al. (2008)
	Raw seeds	14.25	
Myricetin	Dried leaves	5,804	Richter et al. (2003)
isorhamnetin	Dried leaves	30	Bennett et al. (2003)

**FIGURE 2:**
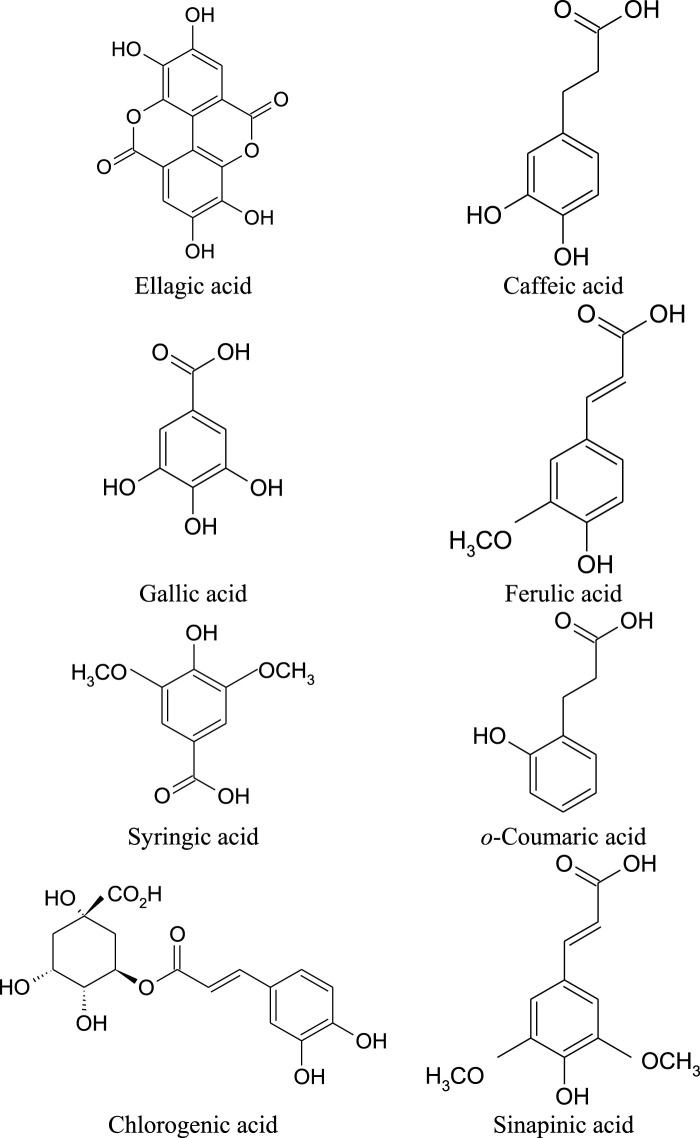
Chemical structures of main phenolic acids found in *M. oleifera*.

Among the leading phenolic acids that are spread mainly in the dried leaves, pods and seeds of *M. oleifera;* gallic acid, ellagic acid, ferulic acid, *o*-coumaric acid, sinapic acid, chlorogenic acid, caffeic acid and syringic acid are listed by the scientists (Figure 2; Table 3) (Yang et al., 2008; Moyo et al., 2011).

**TABLE 3:** The content of phenolic acids in different organs of *M. oleifera* plant.

Metabolites	Raw material	Content (mg/100 g)	References
Total polyphenols	Mature leaves extract	45.81	Sultana and Anwar (2008)
	Immature leaves extract	36.02	
	Dried leaves	1,600–12 200	Ahmed et al. (2016)
	Freeze-dried leaves	1535.6	Moyo et al. (2011)
Gallic acid	Fresh leaves	14.22	Yang et al. (2008)
	Dried leaves	13.72	
	Degreased pods	9.14	
	Raw seeds	6.37	
Chlorogenic acid	Fresh leaves	8.62	
	Dried leaves	6.97	
	Degreased pods	7.44	
	Raw seeds	3.54	
Ellagic acid	Fresh leaves	4.78	
	Dried leaves	2.34	
	Degreased pods	1.53	
	Raw seeds	0.94	
Ferulic acid	Fresh leaves	36.79	
	Dried leaves	33.80	
	Degreased pods	0.75	
	Raw seeds	0.52	
Caffeic acid	Fresh leaves	68.25	
	Dried leaves	65.74	
	Freeze-dried leaves	536	Moyo et al. (2011)
	Degreased pods	32.14	Yang et al. (2008)
	Raw seeds	28.14	
Syringic acid	Fresh leaves	18.16	
	Dried seeds	17.29	
	Degreased pods	0.53	
	Raw seeds	0.44	
*ο*-coumaric acid	Freeze-dried leaves	6,457	Moyo et al. (2011)

Catechins, namely, epicatechin, catechin and pyrocatechin are mainly found in the leaves of *M. oleifera* (Yang et al., 2008; Moyo et al., 2011; Florence, 2014; El-Harairy et al., 2016). Nevertheless, catechin was also detected in its pods and seeds (Table 4) (Yang et al., 2008).

**TABLE 4:** The content of tannins in different morphological organs of *M. oleifera* plant.

Metabolites	Raw material	Content (mg/100 g)	References
Total tannins	Freeze-dried leaves	12	Afuang et al. (2003)
Pyrocatechin	Ethanolic extract from leaves	6.45	Florence (2014)
Catechin	Fresh leaves	18.16	Gopalakrishnan et al. (2016)
	Dried leaves	17.29	
	Degreased pods	3.25	
	Raw seeds	2.96	
Epicatechin	Freeze-dried leaves	5,680	Moyo et al. (2011)

The presence of pyrogallol has been demonstrated in ethanol extracts from the leaves (0.284 mg/100 g) (El-Harairy et al., 2016).

**FIGURE 3:**
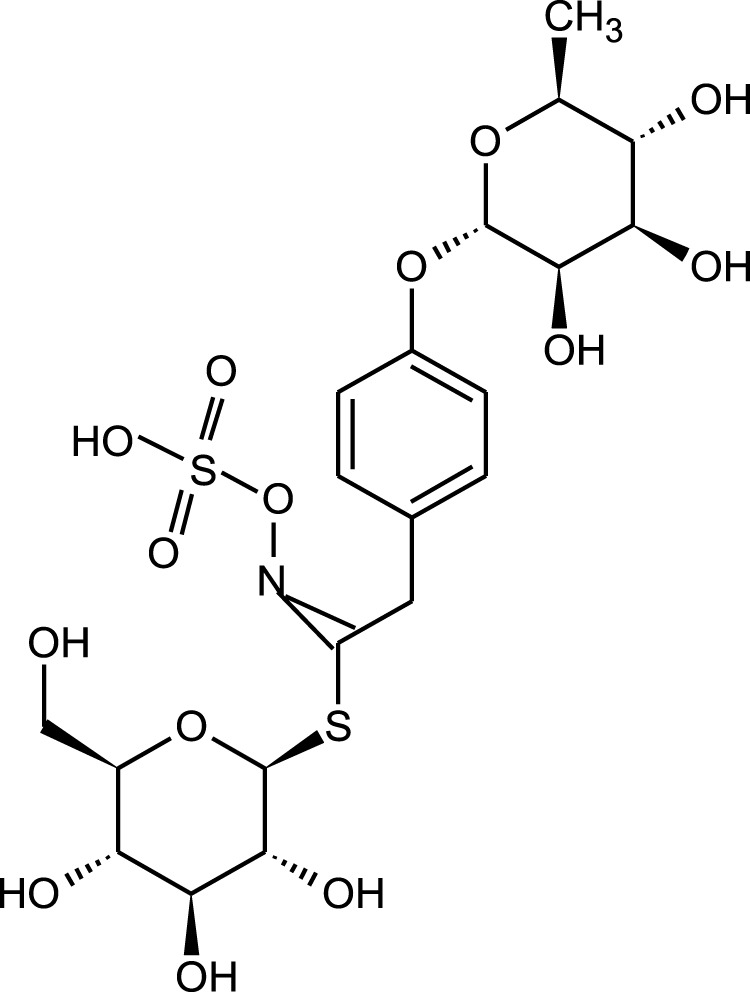
Chemical structure of the glucomoringin found in *M. oleifera.*

**FIGURE 4:**
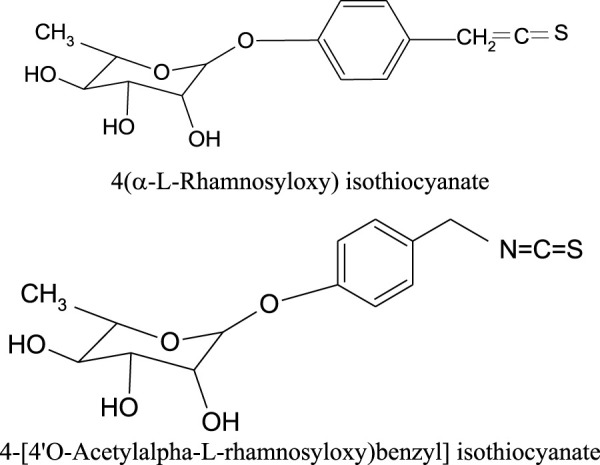
Chemical structures of the isothiocyanates found in *M. oleifera*.

Also, the presence of glucosinolates and their breakdown products, like isothiocyanates, was identified in the roots, seeds and young leaves of *M. oleifera* (Table 5). Large amounts of glucosinolates were found especially in the extracts from vegetatively young leaves. The dominant glucosinolane of *M. oleifera* leaves is 4-*O*-(α-L-rhamnopyranosyloxy)-benzyl glucosinolate, also known as glucomoringin (Figure 3). Moreover, high concentrations of 4-(α-1-rhamnopyranosyloxy)-benzylglucosinolate has been isolated from the seeds, whereas high content of benzylglucosinolate and 4-(α-1-rhamnopyranosyloxy)-benzylglucosinolate was reported in the roots. The concentration of glucosinolanes and isothiocyanins in the leaves of *M. oleifera* is influenced by the maturity and physiological state of the plant (Bennett et al., 2003; Ahmed et al., 2016). Among the isothiocyanates present in the leaves, there is 4-[4′*O*-acetylalpha-L-rhamnosyloxy) benzyl] isothiocyanate, while in seeds 4 (α-L-rhamnosyloxy) isothiocyanate was identified (Figure 4; Table 5) (Eilert et al., 1981; Bennett et al., 2003).

**TABLE 5:** The content of glucosinolates and isothiocyanins in different morphological organs of *M. oleifera* plant.

Metabolites	Raw material	Content (mg/100 g)	References
4-(α-L-rhamnopyranosyloxy)-benzylglucosinolate	Mature leaves	3,390	Bennett et al. (2003)
	Young leaves	5,940	
	Roots	2040	
	Seeds	20 200	
4-hydroxybenzyl	Freeze-dried leaves	590	Ahmed et al. (2016)
4-*O*-(α-L-acetylramnopyranosyloxy)-benzyl isomer 1		690	Bennett et al. (2003)
4-*O*-(α-L-acetylrhhamnopyranosyloxy)-benzyl isomer 2		450	
4-*O*-(α-L-acetylrhamnopyranosyloxy)-benzyl isomer 3		5,040	

**FIGURE 5:**
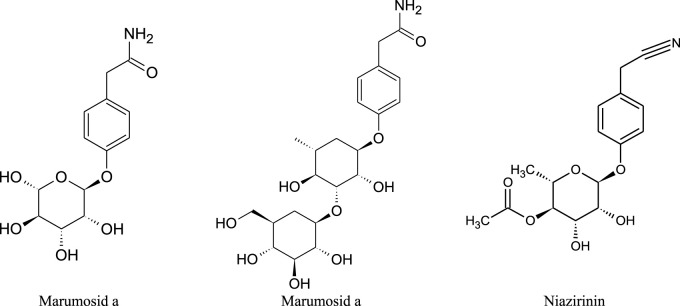
Chemical structures of the alkaloids found in *M. oleifera*.

The presence of alkaloids has been demonstrated in the leaves of *M. oleifera* (Figure 5) such as N,α-L-rhamnopyranosyl vincosidamide, pyrrolemarumin 4″-*O*-α-L-rhamnopyranoside, 4-(α-L-rhamnopyranosyloxy) phenylacetonitrile (niazirin). 4′-Hydroxyphenylethanamide-α-L-rhamnopyranoside (marumoside A) and its 3-*O*-α-D-glucopyranosyl derivative (marmumoside B) and methyl 4-(α-L-rhamnopyranosyloxy)-benzylcarbamate (Ahmed et al., 2016).

**FIGURE 6:**
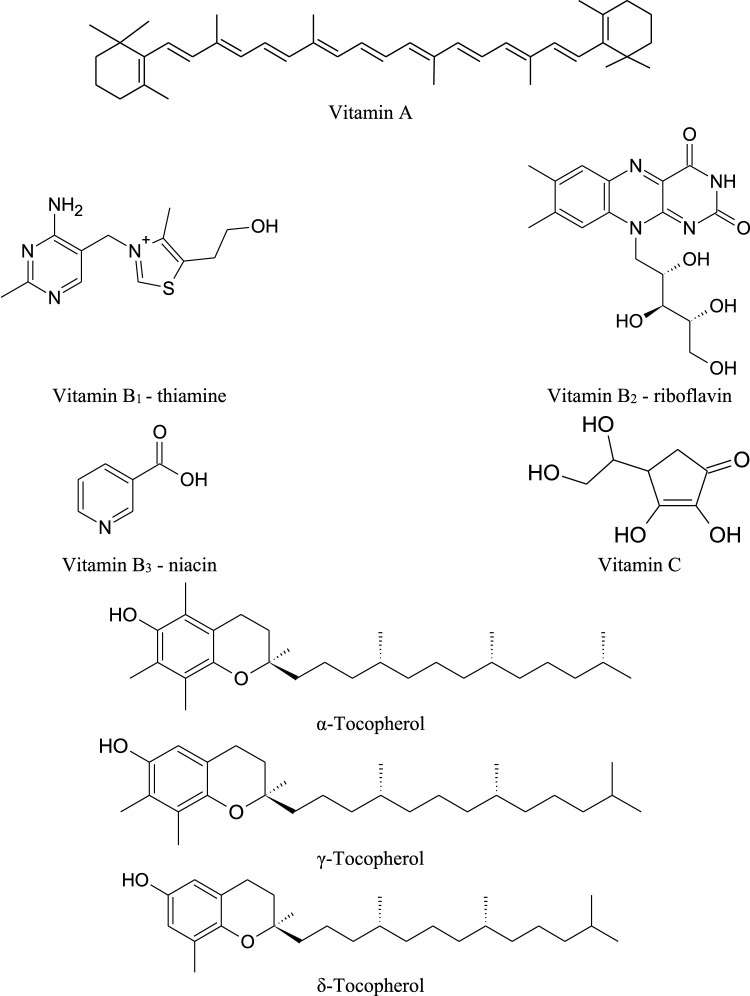
Chemical structures of vitamins found in *M. oleifera*.

Leaves, flowers, pods and seeds of drumstick tree are sources of vitamins. The former showed the presence of vitamins A, B_1_, B_2_, B_3_, C and E (Joshi and Mehta, 2010; Moyo et al., 2011; Zhang, 2011; Saini et al., 2014; Ahmed et al., 2016), the flowers and pods contain vitamin C, and the seeds contain tocopherols (α-tocopherols, γ-tocopherols, δ-tocopherols) (Ijarotimi et al., 2013; Vergara-Jimenez et al., 2017). In addition, the leaves contain carotenoids such β-carotene, 13*-cis*-lutein, 15-*cis-*β-carotene, *trans*-lutein, *trans*-luteoxin i *trans*-zeaxanthin (Figure 6; Table 6) (Joshi and Mehta, 2010; Moyo et al., 2011; Zhang, 2011; Saini et al., 2014).

**TABLE 6:** The content of vitamins in different morphological organs of *M. oleifera* plant.

Metabolites	Raw material	Content	References
Vitamin A	Fresh leaves	11 300—23 000 IU per 100 g fresh weight	Ahmed et al. (2016)
Vitamin B1 (thiamine)		0.01–0.06 mg/100 g	
Vitamin B2 (riboflavin)		0.05–0.17 mg/100 g	
Vitamin B3 (niacin)		0.8–0.82 mg/100 g	
		66.66–143.567 mg/100 g	Mhdawi (2022)
		271 mg/100 g	Saini et al. (2014)
Vitamin C (L-ascorbic acid)	Mature leaves	51.226–140.157 mg/100 g	Mhdawi (2022)
	Flowers	77.50–224.672 mg/100 g	
	Mature flowers	2.3%–9.8%	Taiwo Olagbemide and Alikwe (2015)
	Pods	3.96–8.27 mg/100g	Mhdawi (2022)
	Freeze-dried leaves (Nikaragua)	0.92 mg/100 g	Dhakad et al. (2019)
	Freeze-dried leaves (Indie)	0.84 mg/100 g	
	Freeze-dried leaves (Niger)	0.68 mg/100 g	
	Freeze-dried leaves	1,325.6 mg/100 g	Saini et al. (2014)
	Tray dried leaves (indoor)	590.8 mg/100 g	
	Microwave dried leaves	872.9 mg/100 g	
	Oven dried leaves	545 mg/100 g	
	Leaves dried in the sun	510 mg/100 g	
Vitamin E (α-tocopherol)	Fresh leaves	Około 9.0 mg/100 g	Ahmed et al. (2016)
	Dried leaves	74.45–112.16 mg/100 g	
β-Carotene	Fresh leaves	6.6–6.8 mg/100g	Joshi and Mehta (2010)
	Dried leaves	17.6–39.6 mg/100g	Zhang (2011)
	Freeze-dried leaves	Około 66 mg/100 g	Moyo et al. (2011)
α-Tocopherol	Fresh leaves	36.94 mg/100 g	Saini et al. (2014)
	Freeze-dried leaves	160.2 mg/100 g	
	Tray dried leaves (indoor)	156.2 mg/100 g	
	Microwave dried leaves	135.5 mg/100 g	
	Oven dried leaves	159.6 mg/100 g	
	Leaves dried in the sun	112.3 mg/100 g	
α-Tocopherol	Seed oil (wild variety, Pakistan)	140.5 mg/100 g	Ijarotimi et al. (2013)
	Seed oil (cultivated variety, Pakistan)	134.42 mg/100 g	
	Seed oil (cultivated variety, India)	15.38 mg/100 g	
	Seed oil (cultivated variety, Kenia)	98.82 mg/100 g	
	Seed oil (cultivated variety, Malawi)	131.03 mg/100 g	
γ-Tocopherol	Seed oil (wild variety, Pakistan)	63.18 mg/100 g	
	Seed oil (cultivated variety, Pakistan)	93.70 mg/100 g	
	Seed oil (cultivated variety, India)	4.47 mg/100 g	
	Seed oil (cultivated variety, Kenia)	27.9 mg/100 g	
	Seed oil (cultivated variety, Malawi)	70.43 mg/100 g	
δ-Tocopherol	Seed oil (wild variety, Pakistan)	61.70 mg/100 g	
	Seed oil (cultivated variety, Pakistan)	48 mg/100 g	
	Seed oil (cultivated variety, India)	15.51 mg/100 g	
	Seed oil (cultivated variety, Kenia)	71.16 mg/100 g	
	Seed oil (cultivated variety, Malawi)	53.98 mg/100 g	
*trans*-Luteoxin	Fresh leaves	5.2 mg/100 g	Saini et al. (2014)
	Freeze-dried leaves	18.05 mg/100 g	
	Tray dried leaves (indoor)	17.22 mg/100 g	
	Microwave dried leaves	13 mg/100 g	
	Oven dried leaves	18.03 mg/100 g	
	Leaves dried in the sun	13.22 mg/100 g	
13-*cis*-Lutein	Fresh leaves	2.31 mg/100 g	
	Freeze-dried leaves	6.45 mg/100 g	
	Tray dried leaves (indoor)	10.76 mg/100 g	
	Microwave dried leaves	4.74 mg/100 g	
	Oven dried leaves	9.42 mg/100 g	
	Leaves dried in the sun	9.81 mg/100 g	
*trans*-Lutein	Fresh leaves	36.88 mg/100 g	
	Freeze-dried leaves	94.63 mg/100 g	
	Tray dried leaves (indoor)	87.94 mg/100 g	
	Microwave dried leaves	63.19 mg/100 g	
	Oven dried leaves	81.61 mg/100 g	
	Leaves dried in the sun	80.77 mg/100 g	
*trans*-Zeaxanthin	Fresh leaves	5.46 mg/100 g	
	Freeze-dried leaves	3.7 mg/100 g	
	Tray dried leaves (indoor)	5.42 mg/100 g	
	Microwave dried leaves	16.82 mg/100 g	
	Oven dried leaves	8.31 mg/100 g	
	Leaves dried in the sun	22.55 mg/100 g	
15-*cis*-β-Carotene	Fresh leaves	0.69 mg/100 g	
	Freeze-dried leaves	3.53 mg/100 g	
	Tray dried leaves (indoor)	3.27 mg/100 g	
	Microwave dried leaves	2.5 mg/100 g	
	Oven dried leaves	4.47 mg/100 g	
	Leaves dried in the sun	2.31 mg/100 g	
*trans*-β-Carotene	Fresh leaves	18.27 mg/100 g	
	Freeze-dried leaves	82.13 mg/100 g	
	Tray dried leaves (indoor)	82.33 mg/100 g	
	Microwave dried leaves	65.92 mg/100 g	
	Oven dried leaves	77.79 mg/100 g	
	Leaves dried in the sun	54.42 mg/100 g	
Total carotenoid content	Fresh leaves	68.81 mg/100 g	
	Freeze-dried leaves	208.49 mg/100 g	
	Tray dried leaves (indoor)	206.94 mg/100 g	
	Microwave dried leaves	164.68 mg/100 g	
	Oven dried leaves	199.63 mg/100 g	
	Leaves dried in the sun	183.08 mg/100 g	

Studies have also shown the presence of minerals in the leaves, roots, bark, pods and seeds. The leaves and pods contain zinc, phosphorus, magnesium, manganese, copper, potassium, selenium, sulfur, sodium, calcium and iron. Magnesium, potassium, calcium and iron were found in the root and bark, while zinc, phosphorus, magnesium, potassium, calcium and iron were found in the seeds (Table 7) (Sreelatha and Padma, 2009).

**TABLE 7:** The content of minerals in different morphological organs of *M. oleifera* plant.

Metabolites	Raw material	Content (mg/100 g)	References
Calcium	Fresh leaves	440	Haytowitz and Bhagwat (2010)
	Leaves powder	2003	
	Dried leaves	2,500–3,000	Kashyap et al. (2022)
	Root	28.61	
	Bark	26.41	
	Pod	30	Haytowitz and Bhagwat (2010)
		185	Sreelatha and Padma (2009)
	Degreased seed	371.67	Anwar and Rashid (2007)
	Raw seed	751.67	
Copper	Fresh leaves	1.1	Haytowitz and Bhagwat (2010)
	Pod	3.1	
		0.08	Sreelatha and Padma (2009)
	Leaves powder	0.57	Haytowitz and Bhagwat (2010)
Iron	Fresh leaves	7	
	Leaves powder	28.2	
	Pod	5.3	
		0.36	Sreelatha and Padma (2009)
	Raw seed	5.20	Anwar and Rashid (2007)
	Degreased seed	8.23	
Magnesium	Fresh leaves	24	Haytowitz and Bhagwat (2010)
	Leaves powder	368	
	Pod	24	
		45	Sreelatha and Padma (2009)
	Raw seed	45	Anwar and Rashid (2007)
	Degreased seed	61.67	
Zinc	Fresh leaves	0.6	Sreelatha and Padma (2009)
	Pod	0.45	
	Raw seed	0.05	Anwar and Rashid (2007)
	Degreased seed	0.10	
Phosphorus	Fresh leaves	70	Haytowitz and Bhagwat (2010)
	Leaves powder	204	
	Pod	110	
		50	Sreelatha and Padma (2009)
	Raw seed	635	Anwar and Rashid (2007)
	Degreased seed	273.33	
Potassium	Fresh leaves	259	Haytowitz and Bhagwat (2010)
	Pod	259	
		461	Sreelatha and Padma (2009)
	Raw seed	75	Anwar and Rashid (2007)
	Degreased seed	50	
Sodium	Pod	42	Sreelatha and Padma (2009)
Manganese		0.26	
Selenium		0.7 µg/100 g	
Sulphur		137	Haytowitz and Bhagwat (2010)

**FIGURE 7:**
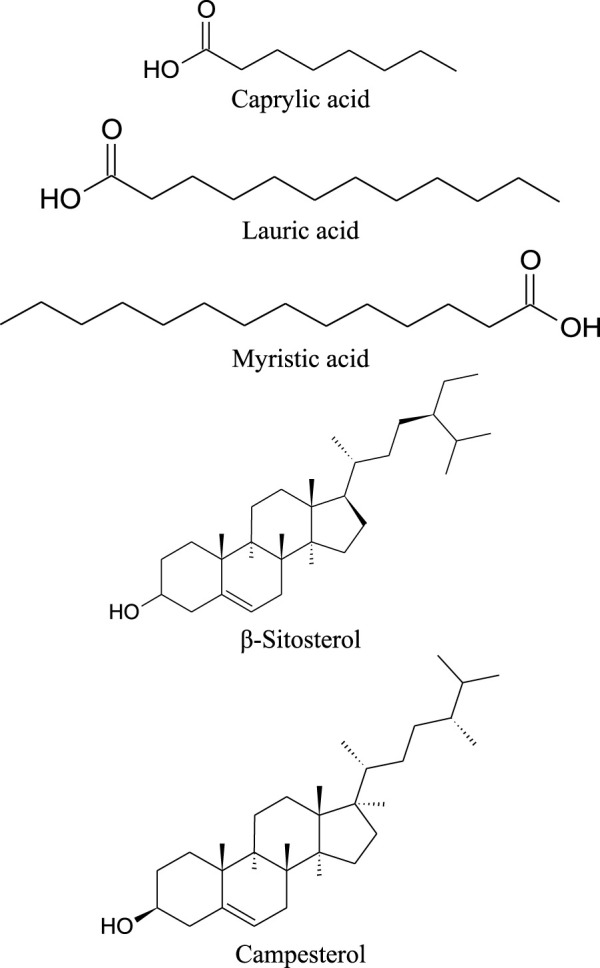
Chemical structures of sterols/oils/fatty acids present in *M. oleifera.*

The leaves of drumstick tree contain arachidic acid, α-linolenic acid, γ-linolenic acid, linolenic acid, myristic acid, oleic acid and palmitic acid (Dai et al., 2020). The seeds were found to contain also other fatty acids, like eicosenoic acid, erucic acid and palmitic acid (Ijarotimi et al., 2013). In addition, the seeds of *M. oleifera* also contain sterols, e.g., campesterol, $\Delta^{5}$ -avenasterol, 24-methylene-cholesterol, campestanol, stigmastanol and clerosterol (Figure 7; Table 8) (Ijarotimi et al., 2013).

**TABLE 8:** The content of sterols/oils/fatty acids in different morphological organs of *M. oleifera* plant.

Metabolites	Raw material	Content	References
Caprylic acid ( $C_{10:0}$ )	Dried leaves	0.07 mg/100 g	Dai et al. (2020)
Lauric acid ( $C_{12:0}$ )		0.58 mg/100 g	
Myristic acid ( $C_{14:0}$ )		3.66 mg/100 g	
Palmitic acid ( $C_{16:0}$ )		11.79 mg/100 g	
	Seed oil (wild variety, Pakistan)	6.45 mg/100 g	Ijarotimi et al. (2013)
	Seed oil (cultivated variety, Pakistan)	6.50 mg/100 g	
	Seed oil (cultivated variety, India)	6.46 mg/100 g	
	Seed oil (cultivated variety, Kenia)	6.04 mg/100 g	
	Seed oil (cultivated variety, Malawi)	5.51 mg/100 g	
	Seed oil	6.45%	
Palmitoleic acid $\left(C_{16:1}\right)$	Seed oil (wild variety, Pakistan)	0.97 mg/100 g	
	Seed oil (cultivated variety, Pakistan)	1 mg/100 g	
	Seed oil (cultivated variety, India)	1.36 mg/100 g	
	Seed oil (cultivated variety, Kenia)	1.46 mg/100 g	
	Seed oil (cultivated variety, Malawi)	1.10 mg/100 g	
Palmitoleic acid ( $C_{16:1}C_{9}$ )	Dried leaves	0.17 mg/100 g	Dai et al. (2020)
Heptadecanoic acid ( $C_{17:0}$ )		3.19 mg/100 g	
Stearic acid ( $C_{18:0}$ )		2.13 mg/100 g	
	Seed oil (wild variety, Pakistan)	5.50 mg/100 g	Ijarotimi et al. (2013)
	Seed oil (cultivated variety, Pakistan)	5.67 mg/100 g	
	Seed oil (cultivated variety, India)	5.88 mg/100 g	
	Seed oil (cultivated variety, Kenia)	4.14 mg/100 g	
	Seed oil (cultivated variety, Malawi)	5.86 mg/100 g	
	Seed oil	5.50%	
Oleic acid $\left(C_{18:1}\right)$	Seed oil (wild variety, Pakistan)	73.22 mg/100 g	
	Seed oil (cultivated variety, Pakistan)	76 mg/100 g	
	Seed oil (cultivated variety, India)	71.21 mg/100 g	
	Seed oil (cultivated variety, Kenia)	73.6 mg/100 g	
	Seed oil (cultivated variety, Malawi)	67.79 mg/100 g	
	Seed oil	73.22%	
Vaccenic acid ( $C_{18:1}C_{7}$ )	Dried leaves	3.96 mg/100 g	Dai et al. (2020)
		0.36 mg/100 g	
Linoleic acid $\left(C_{18:2}\right.$ )	Seed oil (wild variety, Pakistan)	1.27 mg/100 g	Ijarotimi et al. (2013)
	Seed oil (cultivated variety, Pakistan)	1.29 mg/100 g	
	Seed oil (cultivated variety, India)	0.65 mg/100 g	
	Seed oil (cultivated variety, Kenia)	0.73 mg/100 g	
	Seed oil (cultivated variety, Malawi)	0.71 mg/100 g	
Linoleic acid ( $C_{18:2}C_{9.12\left(n-6\right)}$ )	Dried leaves	7.44 mg/100 g	Dai et al. (2020)
		44.57 mg/100 g	
α-Linoleic acid $\left(C_{18:3}\right.$ ) α-Linolenic acid ( $C_{18:3}C_{9.12.15\left(n-3\right)}$ )	Seed oil (wild variety, Pakistan)	0.30 mg/100 g	Ijarotimi et al. (2013)
	Seed oil (cultivated variety, India)	0.18 mg/100 g	
	Seed oil (cultivated variety, Kenia)	0.22 mg/100 g	
	Seed oil (cultivated variety, Malawi)	0.21 mg/100 g	
	Dried leaves	0.20 mg/100 g	Dai et al. (2020)
		1.61 mg/100 g	
Arachidonic acid ( $C_{20:0}$ )	Seed oil (wild variety, Pakistan)	4.08 mg/100g	Ijarotimi et al. (2013)
	Seed oil (cultivated variety, Pakistan)	3 mg/100 g	
	Seed oil (cultivated variety, India)	3.62 mg/100 g	
	Seed oil (cultivated variety, Kenia)	2.76 mg/100 g	
	Seed oil (cultivated variety, Malawi)	3.78 mg/100 g	
	Seed oil	4.08%	
Eicosenoic acid $\left(C_{20:1}\right.$ )	Seed oil (wild variety, Pakistan)	1.68 mg/100 g	
	Seed oil (cultivated variety, Pakistan)	1.20 mg/100 g	
	Seed oil (cultivated variety, India)	2.22 mg/100 g	
	Seed oil (cultivated variety, Kenia)	2.40 mg/100 g	
	Seed oil (cultivated variety, Malawi)	2.60 mg/100 g	
Heneicosanoic acid ( $C_{21:0}$ )	Dried leaves	14.41 mg/100 g	Dai et al. (2020)
		1.24 mg/100 g	
Behenic acid ( $C_{22:0}$ )	Seed oil (wild variety, Pakistan)	6.16 mg/100 g	Ijarotimi et al. (2013)
	Seed oil (cultivated variety, Pakistan)	5 mg/100 g	
	Seed oil (cultivated variety, India)	6.41 mg/100 g	
	Seed oil (cultivated variety, Kenia)	6.73 mg/100 g	
	Seed oil (cultivated variety, Malawi)	6.81 mg/100 g	
	Seed oil	6.16%	
Erucic acid $\left(C_{22:1}\right.$ )	Seed oil (cultivated variety, India)	0.12 mg/100 g	
	Seed oil (cultivated variety, Kenia)	0.14 mg/100 g	
	Seed oil (cultivated variety, Malawi)	0.11 mg/100 g	
Tricosanoic acid ( $C_{23:0}$ )	Dried leaves	0.66 mg/100 g	Dai et al. (2020)
Lignoceric acid ( $C_{24:0}$ )		2.91 mg/100 g	
Total saturated fatty acids		43.31 mg/100 g	
Total monounsaturated fatty acids		4.48 mg/100 g	
Total polyunsaturated fatty acids		52.21 mg/100 g	
Omega-6 fatty acids		7.64 mg/100 g	
Omega-3 fatty acids		44.57 mg/100 g	
Fats	Fresh leaves	1.7 mg/100 g	Zhang (2011)
		1.7 mg/100 g	El-Massary et al. (2013)
	Leaves dried in the sun	6.987 mg/100 g	Zhang (2011)
	Leaves dried in the shade	7.032 mg/100 g	
	Oven dried leaves	7.014 mg/100 g	
	Dried leaves	5.2 mg/100 g	El-Massary et al. (2013)
	Leaf powder	2.3 mg/100 g	
	Roots	10.80 mg/100 g	Pramanik and Islam (1998)
	Bark	17.47 mg/100 g	
	Pods	0.1 mg/100 g	Haytowitz and Bhagwat (2010)
Stigmasterol	Seed oil	18.80%	Ijarotimi et al. (2013)
β-Sitosterol	Seed oil	46.16%	
	Seed oil (wild variety, Pakistan)	46.16 mg/100 g	
	Seed oil (cultivated variety, Pakistan)	46.65 mg/100 g	
	Seed oil (cultivated variety, India)	43.65 mg/100 g	
	Seed oil (cultivated variety, Kenia)	50.07 mg/100 g	
	Seed oil (cultivated variety, Malawi)	47.07 mg/100 g	
Campesterol	Seed oil	17.59%	
$\Delta^{5}$ -Avenasterol	Seed oil	9.26%	
	Seed oil (wild variety, Pakistan)	9.26 mg/100 g	
	Seed oil (cultivated variety, Pakistan)	10.70 mg/100 g	
	Seed oil (cultivated variety, India)	11.61 mg/100 g	
	Seed oil (cultivated variety, Kenia)	8.84 mg/100 g	
	Seed oil (cultivated variety, Malawi)	2.94 mg/100 g	
$\Delta^{7}$ *-* Avenasterol	Seed oil (wild variety, Pakistan)	0.84 mg/100 g	
	Seed oil (cultivated variety, Pakistan)	0.96 mg/100 g	
	Seed oil (cultivated variety, Kenia)	1.11 mg/100 g	
	Seed oil (cultivated variety, Malawi)	0.19 mg/100 g	
24-Methylene-cholesterol	Seed oil (wild variety, Pakistan)	0.99 mg/100 g	
	Seed oil (cultivated variety, Pakistan)	1.49 mg/100 g	
	Seed oil (cultivated variety, India)	0.08 mg/100 g	
	Seed oil (cultivated variety, Kenia)	0.88 mg/100 g	
	Seed oil (cultivated variety, Malawi)	0.96 mg/100 g	
Campesterol	Seed oil (wild variety, Pakistan)	17.95 mg/100 g	
	Seed oil (cultivated variety, Pakistan)	16 mg/100 g	
	Seed oil (cultivated variety, India)	15.29 mg/100 g	
	Seed oil (cultivated variety, Kenia)	15.13 mg/100 g	
	Seed oil (cultivated variety, Malawi)	23.83 mg/100 g	
Campestanol	Seed oil (wild variety, Pakistan)	0.53 mg/100 g	
	Seed oil (cultivated variety Indie)	0.33 mg/100 g	
	Seed oil (cultivated variety, Kenia)	0.5 mg/100 g	
	Seed oil (cultivated variety, Malawi)	0.40 mg/100 g	
$\Delta^{7}$ -Campestanol	Seed oil (wild variety, Pakistan)	0.70 mg/100 g	
	Seed oil (cultivated variety, Pakistan)	0.50 mg/100 g	
Stigmasterol	Seed oil (wild variety, Pakistan)	18.80 mg/100 g	
	Seed oil (cultivated variety, Pakistan)	19 mg/100 g	
	Seed oil (cultivated variety, India)	23.06 mg/100 g	
	Seed oil (cultivated variety, Kenia)	16.87 mg/100 g	
	Seed oil (cultivated variety, Malawi)	17.03 mg/100 g	
Clerosterol	Seed oil (wild variety, Pakistan)	1.70 mg/100 g	
	Seed oil (cultivated variety, Pakistan)	1.95 mg/100 g	
	Seed oil (cultivated variety, India)	1.22 mg/100 g	
	Seed oil (cultivated variety, Kenia)	2.52 mg/100 g	
	Seed oil (cultivated variety, Malawi)	0.62 mg/100 g	
Stigmastanol	Seed oil (wild variety, Pakistan)	0.53 mg/100g	
	Seed oil (cultivated variety, Pakistan)	1 mg/100 g	
	Seed oil (cultivated variety, India)	0.64 mg/100 g	
	Seed oil (cultivated variety, Kenia)	0.86 mg/100 g	
	Seed oil (cultivated variety, Malawi)	0.77 mg/100 g	
28-Isoavenasterol	Seed oil (wild variety, Pakistan)	1.04 mg/100 g	
	Seed oil (cultivated variety, Pakistan)	0.50 mg/100 g	
	Seed oil (cultivated variety, India)	0.25 mg/100 g	
	Seed oil (cultivated variety, Kenia)	1.40 mg/100 g	
	Seed oil (cultivated variety, Malawi)	0.25 mg/100 g	
$\Delta^{7.14}$ -Stigmastanol	Seed oil (wild variety, Pakistan)	0.76 mg/100 g	
	Seed oil (cultivated variety, India)	0.85 mg/100 g	
	Seed oil (cultivated variety, Kenia)	0.44 mg/100 g	

**FIGURE 8:**
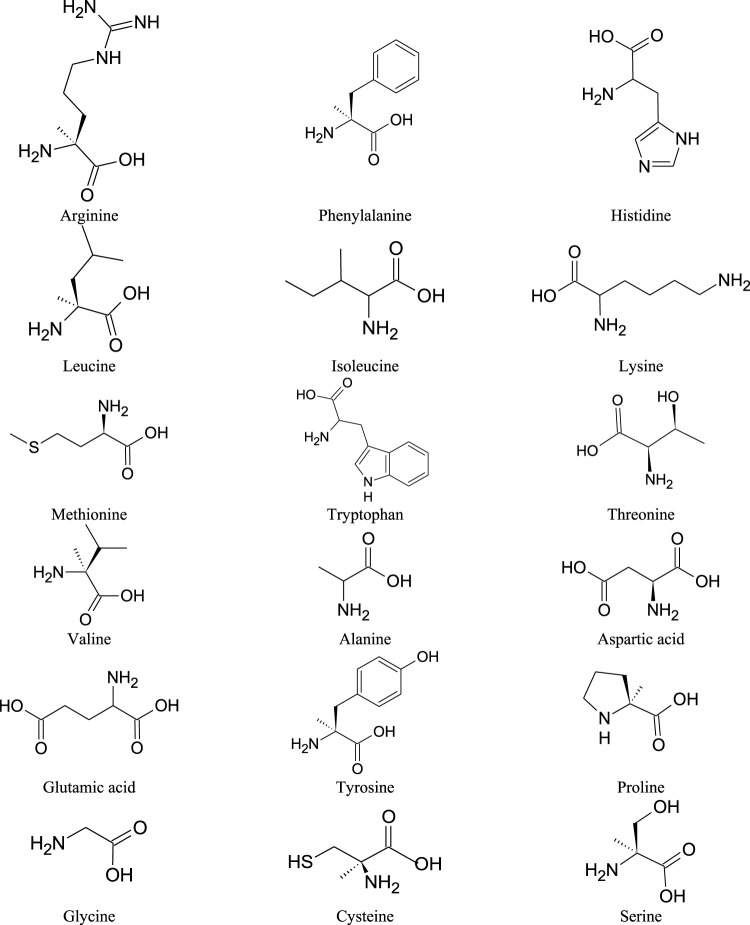
Chemical structures of the amino acids found in *M. oleifera*.

*M. oleifera* seeds are rich sources of high quality protein that contains essential amino acids. The protein content of *M. oleifera* seeds is about 52% (Tang et al., 2021). Similarly, essential amino acids are also found in the leaves and pods of the plant (Yang et al., 2008; Moyo et al., 2011; Dai et al., 2020). They are recommended as a valuable supplement of the diet. The essential amino acids identified in *M. oleifera* include arginine (Arg), phenylalanine (Phe), histidine (His), isoleucine (Ile), leucine (Leu), lysine (Lys), methionine (Met), threonine (Thr), tryptophan (Trp) and valine (Val). Endogenous amino acids include: aspartic acid (Asp), glutamic acid (Glu), alanine (Ala), tyrosine (Tyr), proline (Pro), cysteine (Cys), glycine (Gly), serine (Ser) and hydroxyproline (Hyp) (Figure 8; Table 9) (Yang et al., 2008; Moyo et al., 2011; Dai et al., 2020).

**TABLE 9:** The content of amino acids in different morphological organs of *M. oleifera* plant.

Metabolites	Raw material	Content (mg/100 g)	References
Arginine	Fresh leaves	7.12	Yang et al. (2008)
		402	Haytowitz and Bhagwat (2010)
	Dried leaves	1.78	Dai et al. (2020)
		6.34	Yang et al. (2008)
	Leaf powder	1,325	Haytowitz and Bhagwat (2010)
	Pods	90	
	Degreased pods	8.84	Yang et al. (2008)
	Raw seeds	8.28	Patel et al. (2010)
		12.68	Yang et al. (2008)
	Degummed seed	8.66	Patel et al. (2010)
	Fermented seeds	9.66	
Phenylalanine	Fresh leaves	429	Haytowitz and Bhagwat (2010)
		6.38	Yang et al. (2008)
	Dried leaves	6.29	
		1.64	Moyo et al. (2011)
	Leaf powder	1,388	Haytowitz and Bhagwat (2010)
	Pods	108	
	Degreased pods	3.05	Yang et al. (2008)
	Raw seeds	3.27	Patel et al. (2010)
		3.36	Yang et al. (2008)
	Degummed seed	3.57	Patel et al. (2010)
	Fermented seeds	4.25	
Histidine	Fresh leaves	141	Haytowitz and Bhagwat (2010)
		3.18	Yang et al. (2008)
	Dried leaves	3.04	Yang et al. (2008)
		0.716	Moyo et al. (2011)
	Leaf powder	613	Haytowitz and Bhagwat (2010)
	Pods	27.5	
	Degreased pods	1.72	Yang et al. (2008)
	Raw seeds	1.93	Patel et al. (2010)
		2.74	Yang et al. (2008)
	Degummed seed	2.50	Patel et al. (2010)
	Fermented seeds	2.94	
Isoleucine	Fresh leaves	422	Haytowitz and Bhagwat (2010)
		5.29	Yang et al. (2008)
	Dried leaves	4.59	
		1.177	Moyo et al. (2011)
	Leaf powder	825	Haytowitz and Bhagwat (2010)
	Pods	2.3	Yang et al. (2008)
	Degreased pods	110	Haytowitz and Bhagwat (2010)
	Raw seeds	4.23	Patel et al. (2010)
		3.34	Yang et al. (2008)
	Degummed seed	4.69	Patel et al. (2010)
	Fermented seeds	5.14	
Leucine	Fresh leaves	10.12	Yang et al. (2008)
		623	Haytowitz and Bhagwat (2010)
	Dried leaves	1.96	Moyo et al. (2011)
		8.89	Yang et al. (2008)
	Leaf powder	1950	Haytowitz and Bhagwat (2010)
	Pods	163	
	Degreased pods	4.13	Yang et al. (2008)
	Raw seeds	3.83	Patel et al. (2010)
		5.73	Yang et al. (2008)
	Degummed seed	4.08	Patel et al. (2010)
	Fermented seeds	5.04	
Lysine	Fresh leaves	6.73	Yang et al. (2008)
		288	Haytowitz and Bhagwat (2010)
	Dried leaves	1.637	Moyo et al. (2011)
		5.78	Yang et al. (2008)
	Leaf powder	1,325	Haytowitz and Bhagwat (2010)
	Pods	37.5	Haytowitz and Bhagwat (2010)
	Degreased pods	1.41	Yang et al. (2008)
	Raw seeds	0.13	Patel et al. (2010)
		2.65	Yang et al. (2008)
	Degummed seed	0.363	Patel et al. (2010)
	Fermented seeds	0.405	
Methionine	Fresh leaves	2.12	Yang et al. (2008)
		134	Haytowitz and Bhagwat (2010)
	Dried leaves	0.297	Moyo et al. (2011)
		2.03	Yang et al. (2008)
	Leaf powder	350	Haytowitz and Bhagwat (2010)
	Pods	35	
	Degreased pods	1.44	Yang et al. (2008)
	Fermented seeds	0.41	Patel et al. (2010)
	Raw seeds	2.08	Yang et al. (2008)
		0.31	Patel et al. (2010)
	Degummed seeds	0.35	
Threonine	Fresh leaves	5.15	Yang et al. (2008)
		328	Haytowitz and Bhagwat (2010)
	Dried leaves	1.357	Moyo et al. (2011)
		4.85	Yang et al. (2008)
	Leaf powder	1,188	Haytowitz and Bhagwat (2010)
	Pods	98	
	Degreased pods	1.7	Yang et al. (2008)
	Raw seeds	3.02	Patel et al. (2010)
		2.45	Yang et al. (2008)
	Degummed seeds	3.35	Patel et al. (2010)
	Fermented seeds	3.93	
Tryptophan	Fresh leaves	2.17	Yang et al. (2008)
		127	Haytowitz and Bhagwat (2010)
	Dried leaves	0.486	Moyo et al. (2011)
		2.12	Yang et al. (2008)
	Leaf powder	425	Haytowitz and Bhagwat (2010)
	Pods	20	
	Degreased pods	0.56	Yang et al. (2008)
	Raw seeds	0.69	
Valine	Fresh leaves	6.47	Yang et al. (2008)
		476	Haytowitz and Bhagwat (2010)
	Dried leaves	1.413	Moyo et al. (2011)
		5.79	Yang et al. (2008)
	Leaf powder	1,063	Haytowitz and Bhagwat (2010)
	Pods	135	
	Degreased pods	2.64	Yang et al. (2008)
	Raw seeds	3.78	
		1.08	Patel et al. (2010)
	Degummed seeds	1.25	
	Fermented seeds	1.64	
Aspartic acid	Fresh leaves	10.79	Yang et al. (2008)
	Dried leaves	1.43	Moyo et al. (2011)
		8.89	Yang et al. (2008)
	Degreased pods	3.13	
	Raw seeds	15.70	Patel et al. (2010)
		4.32	Yang et al. (2008)
	Degummed seeds	18.13	Patel et al. (2010)
	Fermented seeds	21.37	
Glutamic acid	Fresh leaves	11.93	Yang et al. (2008)
	Dried leaves	2.53	Moyo et al. (2011)
		10.64	Yang et al. (2008)
	Degreased pods	14.71	
	Raw seeds	17.87	Patel et al. (2010)
		18.76	Yang et al. (2008)
	Degummed seeds	20.23	Patel et al. (2010)
	Fermented seeds	22.46	
Alanine	Fresh leaves	6.72	Yang et al. (2008)
	Dried leaves	3.033	Moyo et al. (2011)
		7.47	Yang et al. (2008)
	Degreased pods	2.87	
	Raw seeds	4.13	
		5.16	Patel et al. (2010)
	Degummed seeds	5.42	
	Fermented seeds	6.29	
Tyrosine	Fresh leaves	4.43	Yang et al. (2008)
	Dried leaves	2.650	Moyo et al. (2011)
		3.96	Yang et al. (2008)
	Degreased pods	1.14	
	Raw seeds	1.75	
		1.97	Patel et al. (2010)
	Degummed seeds	2.09	
	Fermented seeds	2.34	
Proline	Fresh leaves	6.12	Yang et al. (2008)
	Dried leaves	1.203	Moyo et al. (2011)
		5.56	Yang et al. (2008)
	Degreased pods	4.21	
	Raw seeds	5.93	
		2.18	Patel et al. (2010)
	Degummed seeds	2.68	
	Fermented seeds	3.75	
Cysteine	Fresh leaves	1.38	Yang et al. (2008)
	Dried leaves	0.01	Moyo et al. (2011)
		1.23	Yang et al. (2008)
	Degreased pods	3.22	
	Raw seeds	4.59	
		1.68	Patel et al. (2010)
	Degummed seeds	1.79	
	Fermented seeds	2.02	
Glycine	Fresh leaves	6.23	Yang et al. (2008)
	Dried leaves	1.533	Moyo et al. (2011)
		5.58	Yang et al. (2008)
	Degreased pods	3.72	
	Raw seeds	5.34	
		2.37	Patel et al. (2010)
	Degummed seeds	2.63	
	Fermented seeds	3.02	
Serine	Fresh leaves	4.87	Yang et al. (2008)
	Dried leaves	1.087	Moyo et al. (2011)
		4.22	Yang et al. (2008)
	Degreased pods	2.29	
	Raw seeds	2.59	
		2.06	Patel et al. (2010)
	Degummed seeds	3.17	
	Fermented seeds	3.53	
Hydroxyproline	Dried seeds	0.093	Moyo et al. (2011)
Protein	Roots	16.87	Pramanik and Islam (1998)

## 6 Ethnopharmacology

Available sources describe the wide use of *M. oleifera* in traditional Ayurvedic, Chinese (TCM) and African medicine. According to the Ayurvedic pharmacopoeia of India, dried bark and dried seeds of *M. oleifera* have been used in glycosuria and lipid disorders (Government of India Ministry of Ayush, 2016). Among the traditional uses of *M. oleifera* in the Ayurvedic Pharmacopoeia, one can distinguish the following actions: expectorant, antispasmodic, diuretic, stimulant, antiviral, choleretic, tonic, antiseptic, antipyretic, anthelmintic, antiparalytic and analgesic (for sore throat) properties. *M. oleifera* in India was also used in the treatment of asthma and diseases of the liver and spleen. In addition, attention is also drawn to recommending it for the administration in emotional problems or in venereal diseases (Khare, 2007).

*M. oleifera* is listed in the Chinese dictionary of herbal medicine, while in the classical herbal books it is not considered a commonly used herb. In Chinese medicine, the greatest health-promoting properties were attributed to the leaves of the plant that were used in colds (Ross, 2003). The leaves were also attributed to reverse severe malnutrition, rebuild the bone marrow and restore energy by influencing the behavior and maintenance of a positive attitude towards life (Nouman et al., 2014). The leaves were also used for gynecological problems such as vaginal discharge and vaginitis, and in the treatment of anemia (Government of India, Ministry of Ayush, 2016). *M. oleifera* seeds and fruits were recommended in patients with liver diseases, low appetite and flatulence. The root and bark were used to strengthen the functions of the stomach and spleen, aid urination, reduce headaches and dizziness. In addition, they were used in gastrointestinal problems, hypertension, flatulence and skin problems (Government of India, Ministryof Ayush, 2016).

In traditional African medicine, *M. oleifera* species was credited with anti-fatigue action, was used to treat anemia and malnutrition, and for its anthelmintic properties. In Malaysia, poultices of young *M. oleifera* leaves were administered to remove tapeworms. In African countries, the pods are used as an anti-diarrheal agent, in the treatment of dysentery, colitis and gonorrhea, the leaves—to treat inflammation of the glands, headache, and infections, whereas the root, bark and stem—to treat wounds and skin infections. In addition, several examples for the treatment of scurvy, eye pain, digestive ailments were described together with anti-hyaluronidase activity that was useful in the prevention of snake venom from spreading in the bloodstream. Other applications of the bark included the treatment of the upper respiratory tract infections, abortifacient and aphrodisiac properties (Posmontier, 2011).

## 7 Biological activities confirmed by scientific research

The review of the latest studies on the *M. oleifera* biological activity showed that extracts from different parts of the plant and isolated compounds may have different biological activities. A widely tested extract is *M. oleifera* leaf extract, which has both hepatoprotective effects in *in vitro* and *in vivo* tests, as well as antioxidant potential, neuroprotective (*in vivo* and *in vitro* tests), antidepressant (*in vivo* tests) as well as anticancer (*in vivo* and *in vitro* tests) effects. Recent studies have also shown its blood glucose lowering properties (*in vivo* studies), anti-inflammatory properties (*in vitro* studies), wound healing potential (*in vitro* studies), antibacterial and antifungal properties. Research has shown that *M. oleiffera* seed extracts and oil have the potential to be used in cosmetics through anti-aging, photoprotective and anti-psoriasis properties. Plentitude of metabolites of pharmacological significance synthesized by the *M. oleifera* may be used in the treatment of diseases of diverse etymology.

While the research on *M. oleifera* presents promising findings regarding its biological and therapeutic properties, a critical perspective warrants consideration. Firstly, the need for standardized methods across studies could enhance the comparability of results and strengthen the overall reliability of the findings. Additionally, exploring potential side effects or unintended consequences of prolonged use should be a focal point, especially when considering the application of *M. oleifera* extracts in skincare products. Furthermore, it would be valuable for future research to delve deeper into the mechanisms underlying the observed effects. Understanding the specific pathways through which *M. oleifera* components exert their antioxidant influence could provide valuable insights for targeted therapeutic interventions. Moreover, investigating the long-term impact of *M. oleifera* supplementation or application could shed light on its sustainability and safety in extended use. In the context of the *in silico* approach employed in some of the studies, it is crucial to acknowledge the inherent limitations of computational modeling. While molecular docking provides valuable predictions, experimental validation remains paramount to substantiate the proposed mechanisms of action.

Overall, while the current results of research suggest promising possibilities for *M. oleifera* in nutraceutical, cosmetological and pharmaceutical applications, addressing these critical considerations could enhance the scientific robustness and practical implications of these findings.

The biological activities of *M. oleifera* described so far are summarized in the following sections. Table 10 summarizes the latest scientific reports on this issue.

**TABLE 10:** Biological activity of *M. oleifera* confirmed by scientific research.

Properties	Extract/compound	Study description	Results and mechanism of action	References
	Main compound from *M. oleifera* 4-[(α-L-rhamnose-oxy)benzyl] isothiocyanate (MITC) called MITC-12	The study was based on the use of *in vitro* cancer cell lines. The effect of MITC-12 on the U251 (human glioblastoma astrocytoma), A375 (human malignant melanoma), A431 (human squamous carcinoma), HCT-116 (human colon cancer), HeLa (human cervix epitheloid carcinoma) and MDA-MB-231 (human Caucasian breast adenocarcinoma)	MITC-12 significantly inhibited U251 cell proliferation in a time- and dose-dependent manner and reduced the number of EdU-positive cells. MITC-12 induced apoptosis of U251 cells and increased the expression levels of caspase-3 and the Bax:Bcl-2 ratio. MITC-12 inhibits the proliferation of U251 cells, by arresting the cell cycle, activating JNK, and regulating cell cycle-related proteins	Xie et al. (2023)
	Glucomoringin isothiocyanate from *M. oleifera* seeds	The study was based on the use of *in vitro* cancer cell lines. The effect of glucomoringin isothiocyanate on the PC-3 prostate cancer cell line	Glucomoringin isothiocyanate inhibited cell proliferation in the PC-3 cell line (IC50 = 3.5 μg/mL), induced time-dependent G2/M phase arrest and also activates apoptotic genes, including caspase, the tumor suppressor gene (p53), Akt/MAPK, and Bax from the pro-apoptotic Bcl family	Karim et al. (2023)
	Stem and leaves extracts of *M. oleifera*	The effect of stem and leaves extracts of *M. oleifera* isothiocyanate was based on the 4T1 breast cancer cell line derived from the mammary gland tissue of a mouse BALB/c strain	Stem and leaves extracts of *M. oleifera* significantly reduced the proliferation and induced apoptosis of 4T1 cell line. The apoptosis pathway induced by the activation of caspases 9 and -3 and the increase in the Bax/Bcl-2 ratio	Yousefirad et al. (2023)
	*M. oleifera* leaf extracts	The effect of *M. oleifera* leaf extracts was performed in rats with a urethane model of lung cancer	The extracts improved induced changes in the concentrations of glutathione, superoxide dismutase and malondialdehyde. the rats treated with the extract showed a moderate amount of collagen fibers and tumor atrophy and approximately restored normal lung histology	Ibrahim et al. (2023)
Anticancer	Leaf extracts in methanol, ethanol, ethyl acetate and chloroform	The study was based on the use of *in vitro* cancer cell lines. The effect of *M. oleifera* leaf extracts (serial dilutions) on the human cell line B-lymphocytoplasmacytoma-U266B1 was studied. Antitumor activity was measured by neutral red uptake assay	The methanol extract showed the highest activity against U226B1 cells (IC_50_ = 0.32 μg/mL). This is proof of its high anticancer activity	Parvathy and Umamaheshwari (2007)
	Compounds of *M. oleifera*, including rutin, vicenin-2 and glucoside-3-*O*-quercetin	The study aimed to determine the potential therapeutic effect of *M. oleifera* in the treatment of BRCA-1 breast cancer. Molecular docking (computer method) was used to determine the potential pharmacokinetic parameters and drug-like properties. In addition, *in silico* analysis was used using Schrödinger: Molecular and Drug Discovery Suite and SWISS ADME.	The results showed that the compounds contained in *M. oleifera* are so routine, vicenin-2 and glucoside-3-O-quercetin have the highest binding energy in the active site of BRCA-1. It was for rutin −7.522 kcal/mol, for vinicenine-2–6.088 kcal/mol and for glucoside-3-O-quercetin −6.635 kcal/mol. The essential amino acids involved in the protein-ligand interaction after active site analysis include: glycine, leucine, lysine, arginine, and valine	Balogun et al. (2021)
Hepatoprotective	Ethanolic extract of *M. oleifera* seed oil	The effect of an ethanolic extract of *M. oleifera* seed oil on liver markers was evaluated in Wistar rats exposed to dimethyl 2,2-dichlorovinylphosphate (DDVP, known as dichlorvos)	*M. oleifera* seed oil regulated and increased plasma protein levels, reduced elevated levels of liver markers and inflammatory markers. Regenerative effect was demonstrated in histopathological studies	Saka et al. (2022)
	*M. oleifera* leaf extract	The studies was performed on mice with CCl4-induced liver injury	*M. oleifera* leaf extract reduced the elevation of serum alanine aminotransferase (ALT) and aspartate aminotransferase (AST), apoptosis and genotoxicity were alleviated The extract suppressed expression of TLR4 and NF-κB genes, and reduced expression of TLR4 and NF-κB-p65 proteins	Fathy and Mohammed (2021)
	Hydroalcoholic leaf extract	18 albino Wistar rats were studied. They were divided into 3 groups. In group I rats were administered cadmium chloride 200 ppm/kg rats from group II, apart from cadmium chloride, were administered 500 mg/kg of a water-alcoholic extract from *M. oleifera* leaves for 28 days. Group III was the control group	The level of ALT, AST, ALP markers in group treated water-alcoholic extract from *M. oleifera* leaves and controlwas lower than in group treated cadmium chloride. The activity of ALT, AST, ALP enzymes was significantly lower in control group compared to group treated extract. Lipid peroxidation in the liver tissue was significantly higher in group treated extract. There was no significant difference in the concentrations of cadmium in the blood of rats in groups treated cadmium chloride and extract.Elevated serum levels of ALT, ALP, AST could be due to liver damage due to cadmium. The decrease in ALT, AST, ALP could be due to the hepatoprotective effect of *M. oleifera*	Toppo et al. (2015)
	1-*O*-(4-hydroxymethylphenyl)-α-L-rhamnopyranoside (MPG) isolated from *M. oleifera* seed extract	The *in vitro* and *in vivo* study was intended to confirm, whether 1-*O*-(4-hydroxymethylphenyl)-α-L-rhamnopyranoside (MPG) found in *M. oleifera* has hepatoprotective properties. For the study, MPG was isolated from *M. oleifera* seed. Its protection against tetrachloromethane-induced hepatotoxicity was studied in L02 cells and in mice. *M. oleifera* leaf extracts, including ethanol, methanol, aqueous and hexane extracts, were also used	Purified 1-*O*-(4-hydroxymethylphenyl)-α-L-rhamnopyranoside from *M. oleifera* seed extract showed no cytotoxicity. *In vitro* and *in vivo* antihepatotoxicity studies in L02 and mouse ICR cells showed that the compound significantly protects the liver against acute tetrachloromethane-induced hepatotoxicity. This compound alleviated oxidative stress, regulated inflammatory mediators and prevented apoptosis	Sun et al. (2019)
Antioxidant	Se-enriched *M. oleifera* seed protein hydrolysate	The studies was conducted in damaged HepG2 cells	Peptides showed good cellular antioxidant potential, increased cell viability, effective reduction of the accumulation of reactive oxygen species and increasing the activity of superoxide dismutase and catalase	Chen et al. (2023)
	Methanol extracts from *M. oleifera* leaves	The studies was conducted on H_2_O_2_-induced oxidative damage cells	The extracts have a protective effect against H_2_O_2_-induced oxidative damage by increasing cell viability and reducing free radicals. The extract reduced lipid peroxidation and increased the level of glutathione and the activity of antioxidant enzymes, prevented mitochondrial dysfunction by regulating calcium levels and increasing the mitochondrial membrane potential	Gonzalez-Burgos et al. (2021)
	Ethanol extract from *M. oleifera* stems	The study aimed to analyze the effect of *M. oleifera* stem ethanol extract on skin damage caused by oxidative stress caused by hydrogen peroxide and UVB radiation in mice. In addition, the effect of the ethanol extract of the stems on the strengthening of the antioxidant system and the activation of the PPARα receptor *in vitro* and *in vivo* was investigated	Protective effect against oxidative stress of cells *in vitro* (concentration 100–400 μg/mL of extract). Inhibition of UVB-induced damage to the epidermis during topical treatment with a cream with 6% extract	Zhou et al. (2018)
	*M. oleifera* seed oil cream	The study was aimed at evaluating the antioxidant effect of the cream with *M. oleifera* seed oil. The DPPH scavenging test was performed in the study	35.97% neutralization of DPPH by the cream containing 83.33 mg/mL *M. oleifera* seed oil and 4.22% by the cream base	Athikomkulchai et al. (2021)
	*M. oleifera* seed oil	Studies in mice administered *M. oleifera* seed oil for 28 days and then intraperitoneal injected with scopolamine (1 mg/kg) on the same day	The pretreatment with *M. oleifera* seed oil improved the increased levels of TrkB and NF-κB protein expression	Arozal et al. (2022)
	*M. oleifera* dried leaves	The studies demonstrated the effect of a diet supplemented with *M. oleifera* on the spatial memory deficit induced by scopolamine in mice	*M. oleifera* treatment attenuated the loss of spatial memory function by significantly reducing escape latency and significantly increasing crossover frequency with time spent in the platform quadrant, alleviated oxidative stress, restored cholinergic transmission by inhibiting acetylcholinesterase, and maintained neuronal integrity in mouse brain	Onasanwo et al. (2021)
Neuroprotective	Hydroalcoholic leaf extract	The extract was obtained from powdered leaves by cold extraction, it was concentrated under vacuum at room temperature to constant weight. The amount of flavonoids and vitamins was assessed. The experimental animals were thirty male Wistar rats divided into 6 equal groups. Group I received 2 mL/kg of distilled water, group II soybean oil 2 mL/kg, group III *M. oleifera* methanol extract 500 mg/kg, group IV chlorpyrifos 9.8 mg/kg, group V 250 mg/kg *M. oleifera* extract and 30 min later chlorpyrifos, group VI 500 mg/kg *M. oleifera* extract and 30 min later chlorpyrifos. Observations of behavioral and cognitive parameters lasted 9 weeks	After the rats were sacrificed, their brain tissues were collected and the concentration of acetylcholinesterase, superoxide dismutase, glutathione peroxidase, catalase and malondialdehyde concentrations were assessed. Chlorpyrifos-induced oxidative damage and neuronal degeneration have been found. When *M. oleifera* extract was administered, oxidative damage to the brain was less severe, which may be due to the high amount of flavonoids in the *M. oleifera* extract	• Idoga et al. (2018)
	Hydroalcoholic leaf extract	*M. oleifera* leaf extract was tested on male Wistar rats, which were orally administered *M. oleifera* leaf extract at doses of 100, 200 and 400 mg/kg. The results were compared 7 days before and 7 days after the administration of the extract, assessing memory, neuronal density, malondialdehyde (MDA) level and activity of superoxide dismutase (SOD), catalase (CAT), glutathione peroxidase and acetylcholinesterase (AChE) in the hippocampus	The leaf extract improved the animals’ spatial memory and reduced the neurodegeneration of the hippocampus. The level of MDA and (AChE) was reduced, the concentrations of superoxide dismutase and catalase were increased. The authors of the study suggest that the leaf extract may be a potential drug enhancing cognitive functions and neuroprotective, which is probably due to the presence of flavones in the extract	• Sutalangka et al. (2013)
	Seeds and leaves of *M. oleifera*	The aim of the study was to investigate the modulating effect of a diet containing *M. oleifera* seeds and leaves on the brains of rats with streprozotocin-induced diabetes and treated with acarbose. The rats subjected to the experiment were administered intraperitoneally with 0.1 M sodium citrate buffer pH 4.5 containing 60 mg/kg streptozotin. They were fed a diet containing 2% and 4% *M. oleifera* seeds and leaves. Acarbose 25 mg/kg was administered for 14 days by gavage. The animals were divided into 11 groups, each receiving different doses and combinations of test substances	Taking *M. oleifera* orally increases the concentration of antioxidant compounds, decreases the concentration of acetylcholinesterase, butyrylcholinesterase and angiotensin I converting enzyme. These studies suggest the effect of a diet with *M. oleifera* on the prevention of cognitive dysfunction caused by hyperglycemia	Oboh et al. (2018)
Antidepressant	Ethanolic leaf extract	Ethanol extracts from the leaves were tested on Swiss albino mice, which were divided into six groups. Groups I and II were control groups receiving 20 mg/kg fluoxetine. Groups III and IV received ethanol extracts of *M. oleifera* orally at doses of 100 and 200 mg/kg. In groups V and VI animals received combined doses of 100 mg/kg *M. oleifera* ethanol extract and 10 mg/kg fluoxetine and 200 mg/kg *M. oleifera* ethanol extract and 10 mg/kg fluoxetine. After 14 days of treatment, the animals were tested using behavioral models of depression: forced swim test (FST), tail suspension test (TST) and locomotor activity test (LAT)	The study showed significant changes in all tested activities, especially in animals receiving a combined dose of 200 mg/kg *M. oleifera* ethanol extract and 10 mg/kg fluoxetine. The antidepressant effect may be caused by the noradrenergic-serotonergic neurotransmission pathway - a feature of the selective serotonin reuptake inhibitors which is fluoxetine. The exact mechanism of enhancing the effect of fluoxetine with *M. oleifera* extract is not known, it may be related to its anti-inflammatory properties	Kaur et al. (2015)
	*M. oleifera* leaf extract	The studies have demonstrated the antidiabetic nephropathy effect of *M. oleifera* leaf extract administered to rats with streptozotocin-induced diabetic nephropathy	The extract reduced high blood glucose levels, impaired kidney function and oxidative stress parameters in rats. Histological examination of the kidneys showed thickening of the glomerular basement membrane and alleviation of pathological changes. The extract alleviates changes due to the reduction of blood glucose levels, oxidative stress and fibrosis formation by downregulating the expression of TGF-β1 and type IV collagen genes	Thongrung et al. (2023)
	The methanolic extract of *M. oleifera* leaves, methanol extract of *M. oleifera* seeds, and combined leaf and seed extract (2:1 ratio)	The studies investigated the effect of methanolic extract of *M. oleifera* leaves, methanol extract of *M. oleifera* seeds, and combined leaf and seed extract (2:1 ratio) on antidiabetic and antioxidant activities in alloxan-induced diabetes-induced mice. Mice were administered the extracts for 1 and 3 months and compared with appropriate controls	Oral administration of the combined extracts reduced fasting blood glucose levels to normal levels compared to the individual extracts; decrease in cholesterol, triglycerides, creatinine, liver enzymes and oxidative, increase in antioxidant biomarkers	Aljazzaf et al. (2023)
Antidiabetic	Aqueous leaf extract	The aim of the study was to test *M. oleifera* as a potential antidiabetic agent and its effect on immune tolerance. A model of alloxan-induced diabetes in mice was used. Mice treated for diabetes were given 100 mg/kg of *M. oleifera* extract orally. Glucose and insulin levels were tested to calculate insulin resistance. In addition, total antioxidant capacity (TAC) was also determined. Blood urea nitrogen (BUN) and creatinine. Relative concentrations of CD44 proteins were also measured by flow cytometry. CD69 and IFN-γ	In mice with insulin resistance based on a given value of the HOMA-IR insulin resistance index (according to which insulin resistance is diagnosed), its value was 4.5 times higher than in the control group, and in the group treated with *M. oleifera* it was 1.3 times lower. The level of TAC in diabetic mice increases 1.94-fold, and in treated mice it decreases 1.64-fold. In mice treated with *M. oleifera*, blood creatinine and urea nitrogen levels were significantly lower. Better results were obtained for subjects treated with *M. oleifera.* The authors concluded that *M. oleifera* may alleviate insulin resistance. Increase TAC and improve immune tolerance	Sreelatha et al. (2011)
Anti-inflammatory	Methanolic root extract	*M. oleifera* root powder was extracted by soaking in 90% methanol for 72 h, then filtered and the solvent removed by distillation under reduced pressure. The extract was freeze-dried to a gummy paste weighing 19.55 g (from 4.65 kg). The extract was dissolved in an aqueous solution of saline and used for testing. The study used 36 male Wistar rats, which were grouped into 5 groups (6 individuals each). The groups received saline, 100, 250, 500 or 750 mg/kg of *M. oleifera* extract or 10 mg/kg of indomethacin, respectively. Inflammation of the left paw was induced by injecting 0.1 mL of 1% carrageenan in saline	The injection of carrageenan induced intense swelling of the paw, progressing rapidly. At 4–6 h it peaked (90%–100% increase). For doses of 500 mg/kg and 750 mg/kg of *M. oleifera* extract, the effect was reduced by 35%–45%. The lower dose reduced the maximum percentage increase in edema to 55%–65%. The effect at a dose of 660.0 mg/kg resulted in a 50% inhibition of edema growth, which was comparable to the effect of 10 mg/kg of indomethacin. The authors considered the anti-inflammatory effect to be phenolic compounds present in the extract	Ezeamuzie et al. (1996)
	Ethyl acetate leaf extract	The study aimed to evaluate the anti-inflammatory potential and mechanism of cellular action in RAW264.7 cells induced by lipopolysaccharide (LPS). The following leaf extracts were used in the study: chloroform, hexane, butanol and ethyl acetate. The MTT test was also performed to assess cell viability after 24 h. Regardless of the dose, their viability decreased. The following extract concentrations were used in the test: 15.62; 31.25; 62.5; 125; 250; 500 and 1,000 μg/mL	All extracts exhibit significant cytotoxicity at high concentrations, and in particular the chloroform fraction (80% cytotoxicity at 500 and 1,000 μg/mL). All fractions were non-toxic at lower concentrations. Ethyl acetate extract inhibited lipopolysaccharide-induced production of nitric oxide and pro-inflammatory cytokines in macrophages, regardless of concentration. Immunoblot showed that ethylene acetate extract effectively inhibited the expression of inflammatory mediators, including inducible nitric oxide synthase, cyclooxygenase-2 and nuclear factor NF-κB p65 by suppressing the NF-κB signaling pathway. Probably. the phenolic compounds of *M. oleifera* are responsible for the anti-inflammatory effect	Maurya and Singh (2014)
Wound healing	Aqueous leaf extract	The study aimed to develop and formulate a standardized foil dressing with an extract of *M. oleifera* leaves for use in accelerating wound healing. For the purpose of the study, an extract of *M. oleifera* leaves was prepared using absolute ethanol (99.8%) and lower concentrations of ethanol and water. *M. oleifera* leaf extract was subjected to cold maceration in various solvents. After cold extraction, the extract was analyzed (by UHPLC) for the content of bioactive compounds	Acceleration of healing properties in the scratched skin test	Chin et al. (2018)
	Methanolic leaf extract	*In vitro* study using human skin fibroblasts	Increase in the rate of fibroblast proliferation from 17% to 21% for cells treated with low concentrations (0.01 and 0.1 μg/mL) of the extract; increase in cell migration dependent on time (0, 4, 24, 48 h) and dose at concentrations of 0.01 and 0.1 μg/mL. Wound closure in 48 h (0.01 μg/mL extract)	Rodríguez-García et al. (2021)
Anti-psoriatic	Compounds isolated from *M. oleifera* seeds: niazirin, marmumoside A and D-glucoside-3-*O*-β-sitosterol	The study ficused at three compounds identified in *M. oleifera* extracts: niazirin, marmumoside A and D-glucoside-3-*O*-β-sitosterol. Their effect on the expression of Th17-related cytokines was investigated. i.e., IL-12/IL-23 p40. IL-17A. IL-22 and IL-23 p19 using the THP-1 cell line stimulated with LPS. Data were collected at 0.5 h after 2 h for quantification of gene expression. 12-*O*-tetradecanoyl phorbol-13-acetate (TPA) was used in the study. to induce skin lesions that mimic those caused by psoriasis in mice	Attenuation of expression of Th17 essential cytokines; alleviation of TPA-induced skin lesions in mice	Ma et al. (2018)
Antibacterial	Ethanol and aqueous leaf extracts	The study was aimed at evaluating the activity of water and ethanol extracts of *M. oleifera* leaves on the growth of Gram-positive and Gram-negative bacteria by disc diffusion. Paper discs were soaked with 100, 200, 300, 400 µL of extracts. They have been tested against *E. coli, S. aureus, V. parahaemolyticus, E. faecalis, P. aeruginosa*. After 24 h incubation, the zone of inhibition was measured; extracts showing an inhibition zone >13 mm were considered effective	*E. coli, P. aeruginosa* and *S. enteritidis* strains were resistant to *M. oleifera* extracts. The most promising results were obtained with 400 µL of extracts on *S. aureus* (23.3 mm; 25.4 mm), *E. faecalis* (19.4 mm; 17.8 mm), *A. caviae* (23.8 mm; 22.3 mm) and *V. parahemolyticus* (21.9 mm); 20.7 mm) for ethanol and aqueous extracts, respectively	Peixoto et al. (2011)
Antifungal	Ethanol and aqueous leaf extracts	Aqueous and ethanolic (90%) dried leaves were tested by agar plate wells. The antifungal activity against the blank sample was assessed by measuring the zones of inhibition every 24 h. The effect of the extracts was compared with that of amphotericin. The tests were performed on the following fungal strains: *Saccharomyces cerevisiae, Candida albicans* i *Candida tropicalis*	Water and ethanol extracts from the leaves showed the strongest activity against *Saccharomyces cerevisiae* and good antifungal activity against *Candida tropicalis*. No effect has been demonstrated against *Candida albicans*	Patel et al. (2014)
Skin lightening and photoprotective	Cream with *M. oleifera* seed oil and extract from red rice	The study consisted in the preparation of a nourishing herbal cream, also having protective properties against ultraviolet radiation and whitening. Containing *M. oleifera* seed oil, *M. oleifera* leaf powder and red rice extract	Unchanged consistency, smell and color after a month of storage at room temperature; the cream contained 36.4% oleic acid and 0.35% linoleic acid and essential amino acids; the presence of ZnO in the cream was demonstrated	Kumalaningsih and Arwani (2018)
	*M. oleifera* seed extract	The aim of the study was to analyze skin lightening properties on a reconstructed human epidermis model	*M. oleifera* seed extract alone or in combination of *Adansonia digitata* seed oil reduced the melanin content by 21%–27% following 6 days of treatment	Zeitoun et al. (2020)
	*M. oleifera* leaf extract	The aim of the study was to analyze the inhibitory properties of the extract on diphenolase activity of tyrosinase *in vitro*	*M. oleifera* leaf extract effectively inhibited diphenolase activity of tyrosinase *in vitro* (IC50 = 121.3 ± 0.4 μg/mL). Kinetic studies indicated uncompetitive mechanism of tyrosinase inhibitor by *M. oleifera* leaf extract and HPLC analysis combined with computional studies suggested leuteolin as the most probable and potent tyrosinase inhibitor	Hashim et al. (2021)
Anti-aging	*M. oleifera* seed extract with nanoliposomes	The study consisted in the preparation of nanoparticles that increase the penetration and effectiveness of *M. oleifera* seed oil. The parameters assessed are the polydispersity index, determination of particle size, *in vitro* release and *in vivo* skin penetration and *in vitro* protective effect against photoaging	The increase in skin permeability *in vivo* increased from 49.42% to 71.40%	Athira Nair et al. (2020)
			Improvement of *M. oleifera* extract stability	
			Downregulation of MMP-1, MMP-3 and MMP-9 expression	

### 7.1 Anticancer activity

Recent studies have shown that a derivative of the main compound from *M. oleifera* 4-[(α-L-rhamnose oxy) benzyl] isothiocyanate (MITC) called MITC-12 significantly inhibited the growth of U251 (human glioblastoma astrocytoma), A375 (human malignant melanoma), A431 (human squamous carcinoma), HCT-116 (human colon cancer), HeLa (human cervix epitheloid carcinoma) and MDA-MB-231 (human Caucasian breast adenocarcinoma). MITC-12 significantly inhibited U251 cell proliferation in a time- and dose-dependent manner and reduced the number of EdU-positive cells, but was not toxic to normal human gastric mucosa cells (GES-1). MITC-12 induced apoptosis of U251 cells and increased the expression levels of caspase-3 and the Bax:Bcl-2 ratio. These results indicate that MITC-12 inhibits the proliferation of U251 cells, activating JNK, and regulating cell cycle-related proteins (Xie et al., 2023).

Glucomoringin isothiocyanate from *M. oleifera* seeds has an inhibitory effect on cell proliferation in the PC-3 prostate cancer cell line. Glucomoringin isothiocyanate induced time-dependent G2/M phase arrest. Glucomoringin isothiocyanate also activates apoptotic genes, including caspase, the tumor suppressor gene (p53), Akt/MAPK, and Bax from the pro-apoptotic Bcl family (Karim et al., 2023).

Stem and leaves extracts of *M. oleifera* significantly reduced the proliferation and induced apoptosis of 4T1 breast cancer cell line derived from the mammary gland tissue of a mouse BALB/c strain. The results indicated that the apoptosis pathway was likely induced by the activation of caspases 9 and -3 and the increase in the Bax/Bcl-2 ratio (Yousefirad et al., 2023).

Studies of *M. oleifera* leaf extracts in rats with a urethane model of lung cancer showed improved induced changes in the concentrations of glutathione, superoxide dismutase and malondialdehyde. Compared with the control group, rats treated with the extract showed a moderate amount of collagen fibers and tumor atrophy and approximately restored normal lung histology (Ibrahim et al., 2023).

The anticancer effect of *M. oleifera* leaf extracts were tested on a human myeloma cell line (U266B1). Methanol, ethanol, ethyl acetate and chloroform extracts from *M. oleifera* leaves were used in the study. Antitumor activity was measured by the neutral red uptake assay. The methanol extract showed the highest anticancer activity against U226B1 cells (IC_50_ = 0.32 μg/mL) (Table 10) (Parvathy and Umamaheshwari, 2007).

Another study evaluated the effectiveness of *M. oleifera* against the BRCA-1 breast cancer cell line. Molecular docking (computer method), studies of pharmacokinetic parameters and properties similar to other anti-cancer drugs were used to determine the anti-cancer potential. In addition, *in silico* analysis was used using Schrödinger: Molecular and Drug Discovery Suite and SWISS ADME. The results showed that compounds contained in *M. oleifera* such as rutoside, vicenin-2 and glucoside-3-*O*-quercetin have the highest binding energy in the active site of BRCA-1. The essential amino acids involved in the protein-ligand interaction in the active site are: glycine, leucine, lysine, arginine and valine (Table 10) (Balogun et al., 2021).

As shown above, the studies on *M. oleifera* have demonstrated the anticancer potential of this species. Despite the promising results, *in vitro* studies and *in vivo* models require further analysis whether the extracts affect the development of these tumors. Moreover, the molecular docking are the interesting starting point to consider the transmission research into *in vivo* studies.

### 7.2 Hepatoprotective activity

Recent studies have demonstrated the hepatoprotective effects of *M. oleifera* leaf extracts. The effect of an ethanolic extract of *M. oleifera* seed oil on liver markers was evaluated in Wistar rats exposed to dimethyl 2,2-dichlorovinylphosphate (DDVP, known as dichlorvos). *M. oleifera* seed oil regulated and increased plasma protein levels, reduced elevated levels of liver markers and inflammatory markers. Histopathological studies demonstrated the regenerative effect of *M. oleifera* seed oil on dichlorvos-induced liver damage (Saka et al., 2022).

Studies have shown that *M. oleifera* leaf extract reduced the elevation of serum alanine aminotransferase (ALT) and aspartate aminotransferase (AST) levels in mice with CCl4-induced liver injury. CCl4-induced apoptosis and genotoxicity were significantly alleviated by *M. oleifera* leaf extract. Co-administration of the preparation with *M. oleifera* leaf extract modulated the TLR4/NF-κB pathway, which was demonstrated by suppressed expression of TLR4 and NF-κB genes, as well as reduced expression of TLR4 and NF-κB-p65 proteins (Fathy and Mohammed, 2021).

A study on the effect of a hydroalcoholic *M. oleifera* leaf extract on the liver enzymes: alanine aminotransferase (ALT), aspartate aminotransferase (AST) and an enzyme related to the poultry biliary epithelium, alkaline phosphatase (ALP), was performed on rats. The extract was administered for 28 days. Liver damage was induced by administration of cadmium. In the group of animals receiving *M. oleifera* extract, a decrease in the activity of ALT, AST and ALP was demonstrated. Researchers found that the observed decrease in ALT, AST, ALP could be due to the hepatoprotective effect of *M. oleifera* leaf extract, resulting from the presence of quercetin and kaempferol (Table 10) (Toppo et al., 2015).

Another study assessed hepatoprotective properties of 1-*O*-(4-hydroxymethylphenyl)-α-L-rhamnopyranoside (MPG) found in *M. oleifera*. The studies were performed on L02 (human normal hepatocytes) and ICR (mouse inactivated embryonic fibroblasts) with tetrachloromethane-induced hepatotoxicity. *M. oleifera* leaf extracts (ethanol, methanol, aqueous and hexane) were also tested. Purified 1-*O*-(4-hydroxymethylphenyl)-α-L-rhamnopyranoside from the seed extract showed high efficiency. In addition, *in vitro* and *in vivo* antihepatotoxicity studies in mouse L02 and ICR cells showed that this compound significantly protected the liver against acute tetrachloromethane-induced hepatotoxicity. This compound mitigated oxidative stress, regulated the concentration of inflammatory mediators and counteracted apoptosis (Table 10) (Sun et al., 2019).

The exploration of hepatoprotective effects of *M. oleifera* leaf extracts, seed oil, and specific compounds like 1-*O*-(4-hydroxymethylphenyl)-α-L-rhamnopyranoside is intriguing. However, to establish their clinical significance, further research is imperative to validate these findings through comprehensive clinical trials, considering factors such as dosage, long-term effects, and potential side effects in diverse populations. Additionally, a more nuanced understanding of the underlying mechanisms and pathways involved in the hepatoprotective effects of *M. oleifera* compounds would contribute to a more robust assessment of their therapeutic potential in liver-related conditions.

### 7.3 Antioxidant potential

Recent research was conducted on Se-enriched *M. oleifera* seed protein hydrolysate. Peptides showed very good cellular protection and increased cell viability. Moreover, studies have shown effective reduction of the accumulation of reactive oxygen species and increasing the activity of superoxide dismutase and catalase in damaged HepG2 cells (Chen et al., 2023).

Studies have shown that methanol extracts from *M. oleifera* leaves have a protective effect against H_2_O_2_-induced oxidative damage by increasing cell viability and reducing free radicals. In *in vitro* tests, the extract reduced lipid peroxidation and increased the level of glutathione and the activity of antioxidant enzymes. Methanolic extracts from *M. oleifera* leaves also prevented mitochondrial dysfunction by regulating calcium levels and increasing the mitochondrial membrane potential (Gonzalez-Burgos et al., 2021).

The antioxidant activity of *M. oleifera* extracts was evaluated *in vivo* in a mouse model. The study aimed to analyze the effect of ethanol extract from *M. oleifera* stems on skin damage caused by oxidative stress of hydrogen peroxide and UVB radiation. In addition, the effect of the ethanol stem extract on the strengthening of the antioxidant system and the activation of the PPARα receptor *in vitro* and *in vivo* was investigated. It was stated that the extract protected the epidermal cells (keratinocytes) against oxidative stress *in vitro*. Topical treatment of mice with a cream containing *M. oleifera* stem extract inhibited the damage to the epidermis caused by UVB-induced oxidative stress. Then, the phytochemical composition of the extract was analyzed by HPLC and the mechanisms of action were searched using an *in silico* approach - molecular docking. The authors showed that rutoside may be the main component of the extract responsible for the activation of the PPARα receptor and the observed antioxidant effect (Table 10) (Zhou et al., 2018).

The antioxidant power of *M. oleifera* seed oil cream was assessed by Athikomkulchai et al. by the simple DPPH radical scavenging test. The results confirmed that antioxidant properties of the cream dependent on the presence of *M. oleifera* seed oil (Table 10) (Athikomkulchai et al., 2021).

Overall, the collective evidence from these studies suggests that *M. oleifera*, particularly its seed protein hydrolysate, leaf extracts, stem extracts, and seed oil, possesses notable antioxidant potential. These properties could be harnessed for potential therapeutic applications, emphasizing the need for further exploration of *M. oleifera* health-promoting effects.

### 7.4 Neuroprotective activity

In the studies in mice administered with *M. oleifera* seed oil and then intraperitoneally injected with scopolamine have showed that scopolamine increased protein expression of both tropomyosin receptor kinase B (TrkB), a brain-derived neurotrophic factor receptor, and nuclear factor kappa light chain enhancer of activated B cells (NF-κB). However, pretreatment with *M. oleifera* seed oil improved the increased levels of TrkB and NF-κB protein expression (Arozal et al., 2022).

Studies by Onasanwo et al. demonstrated the effect of a diet supplemented with *M. oleifera* on the spatial memory deficit induced by scopolamine. Scopolamine treatment led to loss of spatial memory function during spatial escape platform exploration in mice in the Morris water maze test. Meanwhile, *M. oleifera* treatment attenuated the loss of spatial memory function by significantly reducing escape latency and significantly increasing crossover frequency with time spent in the platform quadrant. Moreover, *M. oleifera* administration alleviated oxidative stress, restored cholinergic transmission by inhibiting acetylcholinesterase, and maintained neuronal integrity in mouse brain (Onasanwo et al., 2021).

Interestingly, *M. oleifera* leaf extract was administered to rats to evaluate its effects on memory mechanisms, neuronal density, malondialdehyde (MDA) levels, superoxide dismutase (SOD) activity, catalase (CAT), glutathione peroxidase (GPx) and acetylcholinesterase (AChE) levels in the hippocampus. The study showed that the treatment improved the spatial memory of the animals and reduced the symptoms of neurodegeneration within the hippocampus. The level of MDA and AChE was reduced, while the expression of SOD and CAT increased. Research results suggest that *M. oleifera* leaf extract, rich in polyphenols, may be a potential drug enhancing cognitive functions and protecting the neuronal cells (Table 10) (Sutalangka et al., 2013).

In another study on rats, the animals were given an extract from dried *M. oleifera* leaves to evaluate neuroprotective effects. When *M. oleifera* extract was administered, oxidative damage to the brain was significantly reduced. The authors of the study attributed this activity to the flavonoids present in the *M. oleifera* extract (Table 10) (Idoga et al., 2018).

The studies Oboh et al. focused on the analysis of the modulating effect of a diet containing *M. oleifera* seeds and leaves on the brains of streptozotocin-induced diabetic rats treated with acarbose. The study showed that *M. oleifera* has an effect on the increase in the concentration of compounds with antioxidant potential, decreases AChE and butyrylcholinesterase levels, as well as angiotensin I converting enzyme. Studies suggest the effect of a diet including *M. oleifera* on the prevention of cognitive dysfunction caused by hyperglycemia (Table 10) (Oboh et al., 2018).

The studies investigating the neuroprotective effects of *M. oleifera* in various experimental models provide valuable insights into its potential cognitive benefits. However, critical considerations include the need for more standardized protocols and diverse experimental settings to establish the consistency and generalizability of the observed effects across different neurological conditions. Additionally, future research should delve into the specific mechanisms through which *M. oleifera* influences neurobiology, ensuring a more comprehensive understanding of its potential therapeutic applications in cognitive health.

### 7.5 Antidepresant activity

Ethanol extracts from *M. oleifera* leaves were tested on mice using behavioral models of depression: in the forced swim test (FST), tail suspension test (TST) and locomotor activity test (LAT). The study showed significant changes in all tested activities, especially in the group receiving a combined dose of 200 mg/kg *M. oleifera* ethanol extract and 10 mg/kg fluoxetine. The antidepressant effect may be related to the effect on the noradrenergic-serotonergic neurotransmission pathway. The exact mechanism by which the effect of fluoxetine is enhanced by *M. oleifera* extract has not been indicated yet but may be related to its anti-inflammatory properties (Table 10) (Kaur et al., 2015).

While the study on the antidepressant effects of *M. oleifera* leaves is intriguing, the absence of a clear elucidation of the synergistic mechanism between the extract and fluoxetine raises questions about the precise interactions that enhance the antidepressant effect. Further research elucidating the specific pathways and molecular targets influenced by *M. oleifera* in conjunction with fluoxetine would provide a more comprehensive understanding of its potential role in augmenting antidepressant treatment.

### 7.6 Antidiabetic activity

Recent studies have demonstrated the antidiabetic nephropathy effect of *M. oleifera* leaf extract administered to rats with streptozotocin-induced diabetic nephropathy. Administration of the extract reduced high blood glucose levels, impaired kidney function and oxidative stress parameters in rats. Histological examination of the kidneys showed thickening of the glomerular basement membrane and alleviation of pathological changes. Studies have shown that *M. oleifera* leaf extract alleviates changes due to the reduction of blood glucose levels, oxidative stress and fibrosis formation by downregulating the expression of TGF-β1 and type IV collagen genes (Thongrung et al., 2023). Aljazzaf et al. investigated the effect of methanolic extract of *M. oleifera* leaves, methanol extract of *M. oleifera* seeds, and combined leaf and seed extract on antidiabetic and antioxidant activities in alloxan-induced diabetes-induced mice. Oral administration of the combined extracts significantly reduced fasting blood glucose levels to normal levels compared to the individual extracts; in addition, a significant decrease in cholesterol, triglycerides, creatinine, liver enzymes and oxidative markers was observed, with a simultaneous increase in antioxidant biomarkers (Aljazzaf et al., 2023).

The model of alloxan-induced diabetes in mice was used to study the anti-diabetic properties of *M. oleifera* extracts. The level of TAC (Total Antioxidant Capacity) in diabetic mice increased 1.94-fold, and in those taking the extract it decreased 1.64-fold. In mice treated with *M. oleifera*, the levels of creatinine and urea nitrogen in the blood were significantly lower than in animals that did not take the extract. Based on the presented data, the authors conclude that *M. oleifera* leaf extract may alleviate insulin resistance, increase TAC and improve immune tolerance in people with diabetes (Table 10) (Sreelatha et al., 2011).

The ethanol leaf extract of *M. oleifera* was also evaluated in the pancreatic α-amylase enzymatic inhibition test (Table 10) (Chigurupati et al., 2022).

While the studies highlight the potential antidiabetic effects of drumstick tree leaf extract in various experimental models, critical considerations include the need for more standardized protocols and diverse experimental settings to establish the consistency and generalizability of the observed effects across different diabetes-induced conditions. Additionally, a comprehensive understanding of the underlying mechanisms and pathways through which *M. oleifera* influences antidiabetic outcomes is crucial for validating its therapeutic potential in managing diabetic nephropathy and insulin resistance.

### 7.7 Anti-inflammatory activity

A study of the anti-inflammatory effect of *M. oleifera* root extracts was performed on rats in which the left paw was inflamed by injecting 0.1 mL of 1% carrageenan in saline. *M. oleifera* extract, depending on the concentration, reduced swelling by 35%–45% (Table 10) (Ezeamuzie et al., 1996).

Another group evaluated the anti-inflammatory effects of *M. oleifera* and its potential use in treating urinary tract infections in a human study. The first group of study participants received *M. oleifera* bark extract and medications and the other group was given only modern drugs. The study was conducted for a period of 12 weeks. In the group treated with drugs and *M. oleifera*, 66.67% of patients were cured. In the case of drugs administered alone the rate of 46.67% only was achieved. The authors of the study emphasize that the study needs to be replicated on a larger scale and over a longer period of time. In addition, they pointed out that polyphenolic compounds are responsible for the anti-inflammatory effect of *M. oleifera* (Table 10) (Maurya and Singh, 2014).

In another study, four types of *M. oleifera* leaf extracts were analyzed: chloroform, hexane, butanol and ethyl acetate. The study aimed to evaluate the anti-inflammatory potential and mechanism of cellular action in RAW264.7 cells induced by lipopolysaccharide (LPS). In the MTT (3-(4,5-dimethylthiazol-2-yl)-2,5-diphenulotetrazolium) assay to measure cell viability after 24 h of treating cells with extracts. Regardless of the dose, their viability decreased. All fractions were non-toxic at lower concentrations. A cellular cytotoxicity test was performed, which showed that the extract was not cytotoxic to macrophages at a concentration of up to 200 μg/mL. Acetate extract inhibited LPS-induced production of nitric oxide and pro-inflammatory cytokines in macrophages, regardless of concentration. It was the most effective extract. Immunoblotting showed that the acetate extract effectively inhibited the expression of inflammatory mediators, including inducible nitric oxide synthase (NOS), cyclooxygenase-2 (COX-2) and nuclear factor (NF)-κB p65 by suppressing the NF-κB signaling pathway (Table 10) (Arulselvan et al., 2016). While the anti-inflammatory effects of *M. oleifera* root extracts in the carrageenan-induced rat paw model are promising, the study lacks detailed insights into the specific mechanisms underlying this reduction in swelling, leaving questions about the precise pathways and mediators involved. Similarly, the human study on *M. oleifera* bark extract’s potential for treating urinary tract infections provides interesting preliminary results, but the short duration and limited scale of the study warrant caution in drawing definitive conclusions, emphasizing the need for more extensive and prolonged investigations. Table 10 presents a comparison of the key research findings regarding the anti-inflammatory properties of this plant.

### 7.8 Wound healing potential

A study by Chin et al. aimed to develop and formulate a standardized foil dressing with *M. oleifera* leaf extract for use accelerating wound healing. For the purpose of the study, an extract of *M. oleifera* leaves was prepared using absolute ethanol (99.8%) and lower concentrations of ethanol and water. *M. oleifera* leaf extract was subjected to cold maceration in various solvents. After cold extraction, the extract was analyzed (by UHPLC) for the content of bioactive compounds. It was found that the richest was the aqueous extract. Subsequently, tests were carried out on scratched skin. The best healing properties were shown by the aqueous extract of *M. oleifera* leaves. According to the authors of the study, the healing properties of *M. oleifera* were probably due to the high content of bioactive compounds: chlorogenic acid, gallic acid, quercetin, kaempferol, rutoside and vicenin-2 (Table 10) (Chin et al., 2018).

In a study by Rodríguez-García et al. methanol extracts from *M. oleifera* leaves were tested. The research was carried out using human skin fibroblasts. A significant increase in the rate of fibroblast proliferation from 17% to 21% was observed. When cells were treated with low concentrations (0.01 and 0.1 μg/mL) of methanolic extract from *M. oleifera* leaves. Cell proliferation was dependent on the concentration of the extract; for the concentration of 500 μg/mL, the best effects of inhibition of proliferation were found. In the next step, a 1.5 mm wound was made in the fibroblast cell monolayer and incubated for 48 h at a specific concentration of the extract. Cells incubated with *M. oleifera* extract at concentrations of 0.01 and 0.1 μg/mL showed time (0, 4, 24, 48 h) and dose-dependent increase in migration. The extract with a concentration of 0.01 μg/mL turned out to be the most effective and closed the wound within 48 h. The study showed that 0.01 μg/mL methanol extract from *M. oleifera* leaves determines the highest proliferation and migration (70%) of human skin fibroblasts *in vitro*. In the studies, no toxicity of the extract was observed on fibroblasts. According to the researchers, flavonoids and other biologically active compounds were responsible for the obtained effects. Chromatographic analysis of the extract showed the presence of chlorogenic acid, myricetin 3-*O*-xylopyranoside, neohesperidin, rutoside, isoquercetin, kaempferol, quercetin and vicenin-2 (Table 10) (Rodríguez-García et al., 2021). The efforts by Chin et al. (2018) to develop a standardized foil dressing with *M. oleifera* leaf extract for wound healing show promise, especially with the identification of bioactive compounds like chlorogenic acid, gallic acid, quercetin, kaempferol, rutoside, and vicenin-2. However, the clinical relevance of these findings requires further validation through comprehensive clinical trials to establish the efficacy and safety of the proposed dressing in accelerating wound healing in real-world scenarios. Similarly, Rodríguez-García et al. (2021) study on methanol extracts from *Moringa* leaves demonstrates increased fibroblast proliferation and migration, yet the concentration-dependent effects and mechanisms underlying these observations warrant thorough exploration. The absence of observed toxicity is encouraging, but the translatability of these *in vitro* findings to human wound healing processes necessitates additional research, emphasizing the need for rigorous clinical investigations to ascertain the practical applicability of *M. oleifera* in wound healing.

### 7.9 Antipsoriatic activity

Ma et al. investigated the potential use of *M. oleifera* in the treatment of psoriasis. In the pathogenesis of psoriasis, Th17 lymphocytes with the participation of cytokines IL-23 and p19 play a key role. The anti-inflammatory effects of the bioactive components of *M. oleifera* have not been well elucidated and therefore limited information is available on its potential clinical use. Researchers have identified in the seed three compounds potentially important in the treatment of psoriasis, namely, niazirin, marmumoside A and D-glucoside-3-*O*-β-sitosterol. Subsequently, their effect on the expression of Th17-related cytokines was investigated. i.e., IL-12/IL-23 p40. IL-17A. IL-22, and IL-23 p19 using the THP-1 cell line stimulated with LPS (lipopolysaccharide). Data were collected at 0.5 h and 2 h for quantification of gene expression. The study used 12-*O*-tetradecanoyl phorbol-13-acetate (TPA) to induce skin lesions mimicking those caused by psoriasis in mice. Increased expression of pro-inflammatory cytokines associated with psoriasis was found. In the study, niazirin, marumoside A and D-glucoside-3-*O*-β-sitosterol, isolated from *M. oleifera*, attenuated the expression of cytokines essential for Th17. They also alleviated TPA-induced skin lesions in mice. This is the first report on the effectiveness of *M. oleifera* in psoriasis-like lesions *in vivo* (Table 10) (Ma et al., 2018). While Ma et al.'s investigation into the potential use of drumstick tree in treating psoriasis is promising, the limited understanding of the anti-inflammatory effects of its bioactive components raises questions about the depth of knowledge in this area. The quantitative analysis of the plant is noteworthy, but further research is crucial to elucidate the specific mechanisms and clinical implications of these components in psoriasis treatment. The study’s use of a mouse model to mimic psoriasis-like lesions is a significant step, but the translatability of these findings to human psoriasis requires careful consideration, emphasizing the need for more comprehensive clinical investigations to establish the potential clinical utility of *M. oleifera* in psoriasis management.

### 7.10 Antibacterial activity

The antibacterial activity of *M. oleifera* was assessed using aqueous and ethanol leaf extracts and the Gram-positive (*Enterococcus faecalis* and *Staphylococcus aureus*) and Gram-negative (*Aeromonas caviae, E. coli, Pseudomonas aeruginosa, Salmonella enteritidis* and *Vibrio parahaemolyticus*) bacterial strains*. E. coli, P. aeruginosa* and *S. enteritidis* were resistant to tested *M. oleifera* extracts. The most promising results were obtained in an assay where the extracts were used against *S. aureus* (inhibition zones: 23.3 mm; 25.4 mm), *E. faecalis* (inhibition zones: 19.4 mm; 17.8 mm), *A. caviae* (inhibition zones: 23.8 mm; 22.3 mm) and *V. parahemolyticus* (inhibition zones: 21.9 mm; 20.7 mm), for ethanol and aqueous extracts, respectively (Table 10) (Peixoto et al., 2011).

*M. oleifera* seed powder was subjected to an antibacterial test against *Salmonella* sp., *S. aureus* and *E*. *coli* strains. In the antibacterial test, *M. oleifera* powder was effective against each strain. The MIC values were 99.4% for *Salmonella sp*., 78.8% for *E. coli* and 57.3% for *S. aureus*, respectively (Table 10) (Aboagye et al., 2021).

The assessment of *M. oleifera*antibacterial activity is valuable, yet the observed resistance of *E. coli, P. aeruginosa*, and *S. enteritidis* to the tested extracts raises questions about the broad-spectrum efficacy. Further exploration into the specific mechanisms underlying the observed antibacterial effects and potential variations in response among bacterial strains would contribute to a more nuanced understanding of *M. oleifera* applicability in combating bacterial infections. Additionally, the antibacterial test using *M. oleifera* seed powder presents promising results, but further investigations should address the concentration-dependent effects and consider potential variations in efficacy against different bacterial strains to ensure a comprehensive evaluation of its antibacterial potential.

### 7.11 Antifungal activity

The antifungal effect of aqueous and 90% ethanol extracts from dried leaves of *M. oleifera* was carried out against the following fungal strains: *Saccharomyces cerevisiae*, *Candida albicans* and *Candida tropicalis*. The extracts showed the strongest activity against *S. cerevisiae*, good antifungal activity against *C. tropicalis* and no activity against *C. albicans* (Table 10) (Patel et al., 2014). While the investigation into the antifungal effect of *M. oleifera* leaf extracts against various strains is noteworthy, critical considerations should address the specificity of the observed activity. Understanding the underlying mechanisms and potential variations in response among different fungal strains is crucial for assessing the practical applicability of these extracts. Additionally, further research should explore the safety and potential side effects of *M. oleifera* extracts, especially when considering their use as antifungal agents, to ensure a comprehensive evaluation of their efficacy and potential limitations.

### 7.12 Skin lightening and photoprotective potential

*M. oleifera* seed oil, due to the presence of essential fatty acids, has become the subject of research to create a nourishing herbal cream that also has ultraviolet radiation protection and skin brightening properties. In order to develop the cream, in addition to seed oil, *M. oleifera* leaf powder and red rice extract were also used. The tests showed that the prepared cream contained 36.4% oleic acid and 0.35% linoleic acid, and also contained essential amino acids. Fortification of red rice showed the presence of ZnO in the cosmetic, which has a protective effect against UV radiation. On the basis of the organoleptic assessment of the cream’s stability at room temperature, it was found that within 1 month the cream still has the correct consistency, smell and color. Studies show that environmental factors such as the Sun do not adversely affect the consistency of cosmetics with *M. oleifera* seed oil (Table 10) (Kumalaningsih and Arwani, 2018).

Skin lightening properties of *M. oleifera* seed extract were also proven using reconstructed human epidermis model. *M. oleifera* alone or in combination of *Adansonia digitata* seed oil reduced the melanin content by 21%–27% following 6 days of treatment. Obtained results was comparable with kojic acid, a substance currently used in skin lightening cosmetics (Zeitoun et al., 2020).

Skin-lightening potential was also shown for *M. oleifera* leaf extract which effectively inhibited diphenolase activity of tyrosinase *in vitro* (IC50 = 121.3 μg/mL). Kinetic studies indicated uncompetitive mechanism of tyrosinase inhibitor by *M. oleifera* leaf extract and HPLC analysis combined with computational studies suggested luteolin as the most probable and potent tyrosinase inhibitor (Hashim et al., 2021).

However, the critical evaluation should extend to long-term stability assessments and potential interactions between ingredients, especially under varied environmental conditions. Additionally, while promising, the skin-lightening properties demonstrated by *M. oleifera* seed and leaf extracts should prompt further investigations into their safety and long-term effects on skin health, addressing concerns related to prolonged use and potential side effects in diverse skin types.

### 7.13 Anti-aging activity

Isothiocyanates obtained from *M. oleifera* have anti-aging properties (Athira Nair et al., 2020). However, due to low solubility in water, easy degradation and difficult bioavailability, the use of preparations consisting of *M. oleifera* solely does not bring the expected results. Wang et al. investigated the combination of *M. oleifera* seed extract with nanoliposomes. Flexible nanoliposomes are effectively used as biocompatible polymers used, among others, for transdermal drug delivery. The study consisted of the preparation of amphiphilic hyaluronic acid conjugated with ceramide in order to provide an extract from *M. oleifera* seeds. The combined nanoparticles of *M. oleifera* extract, hyaluronic acid and ceramide have been tested in many aspects, such as polydispersity index, determination of particle size, *in vitro* release and *in vivo* skin penetration and *in vitro* photoaging protective effect. Due to the use of nanoparticles, the effectiveness of *M. oleifera* has significantly increased. The effectiveness of introducing the *M. oleifera* seed extract into the liposomes increased the effectiveness of the extract from 62.54% to 70.67%. and the size of the nanoparticles decreased from 266.1 nm to 192.8 nm. Good results were also observed in an *in vivo* skin permeation study, which showed an increase in permeability from 49.42% to 71.40%. In addition, the stability of the *M. oleifera* extract was improved (Wang et al., 2022). The nanoparticles also improved the action of antioxidant enzymes in the skin by scavenging free radicals that are induced by UVB. The result is skin protection and a reduction in the expression of matrix metalloproteases MMP-1, MMP-3 and MMP-9 activated in the photoaging process. Flexible nanoliposomes effectively improved the penetration of *M. oleifera* seed extract, however, the authors of the study concluded that more research should be conducted to confirm the thesis about their use in the prevention and therapy of photoaging (Table 10) (Athira Nair et al., 2020).

Despite the promising, anti-aging potential of *M. oleifera*, there are inherent challenges of low solubility, degradation, and poor bioavailability of the extract components necessitate innovative approaches. The strategy by combining *M. oleifera* seed extract with nanoliposomes seems to be a good solution for this issue. The nanoparticles also enhanced antioxidant enzyme activity, offering protection against UVB-induced free radicals and reducing the expression of matrix metalloproteases linked to photoaging. While these results are promising, the authors rightly call for further research to validate the application of these nanoliposomes in the prevention and therapy of photoaging.

## 8 *M. oleifera* as dietary supplement

The chemical composition of *M. oleifera*, and especially extracts from the leaves of the plant, determines their use in medicine. This plant has anti-inflammatory properties, antioxidant, hepatoprotective, neuroprotective and anticancer. Studies also show an anti-aging and wound healing effects. In addition, it can be used to support the treatment of pain, depression, psoriasis and diabetes. The bactericidal properties of the plant are also of great importance. A number of studies prove the antimicrobial activity of *M. oleifera* (Peixoto et al., 2011; Aboagye et al., 2021). There are many *M. oleifera* supplements on the market today (*e.g.*, Swanson Moringa oleifera, Yango Moringa, Vitama Nature Moringa, Jiva Botanicals Moringa, Natgrown Moringa oleifera leaf).

## 9 Application in cosmetic industry

A summary description of the functions of individual ingredients and raw materials obtained from *M. oleifera*, approved for the production of cosmetics for the indicated purposes according to CosIng (Cosmetic Ingredient Database) (European Commission Cosing, 2020) is presented in Table 11. The oil and extracts obtained from *M. oleifera* are present in preparations produced, in Poland, Great Britain, Germany, United States of America, Italy, France, Bulgaria and Cambodia. They include face creams, eye creams, body and hair care products. In cosmetics, they provide antioxidant, regenerating or anti-aging properties (Athira Nair et al., 2020; Wang et al., 2022).

**TABLE 11:** The use of *M. oleifera* according to CosIng (Cosmetic Ingredient Database CosIng, 2023).

INCI name	Description	Activity
Hydrogenated *Moringa oleifera* Seed Oil	Product obtained from the controlled hydrogenation of *M. oleifera*	• skin conditioning—emollient
		• skin conditioning
Hydrolyzed *Moringa oleifera* Seed Extract	The hydrolysate of *M. oleifera* seed extract (q.v.) derived by acid. Enzyme or other method of hydrolysis	• skin conditioning
*Lactobacillus*/*Moringa oleifera* Leaf Extract Ferment Filtrat	The filtrate of the product obtained by the fermentation *M. oleifera* leaf extract by the microorganism. *Lactobacillus*	• skin conditioning - emollient
*Moringa oleifera* Bark Extract	The extract of the bark of *M. oleifera*	• skin conditioning
*Moringa oleifera* Callus Extract	The extract of the callus of *M. oleifera*	• antioxidant
		• hair conditioning
		• skin protecting
*Moringa oleifera* Fruit Powder	The powder obtained from the dried. Ground fruit of *M. oleifera*	• abrasive
*Moringa oleifera* Leaf Powder	The powder obtained from the dried. Ground leaves of *M. oleifera*	• antimicrobial
		• antioxidant
		• antiplaque
		• hair conditioning
		• oral care
		• skin conditioning
*Moringa oleifera* Leaf Extract	Extract of the leaves of *M. oleifera*	• skin conditioning
*Moringa oleifera* Leaf Water	The aqueous solution of the steam distillate obtained from the leaves of *M. oleifera*	• skin conditioning
*Moringa oleifera* Seed Extract	The extract of the seeds of *M. oleifera*	• skin conditioning
*Moringa oleifera* Seed Oil	The oil expressed from the seeds of *M. oleifera*	• skin conditioning—emollient
		• skin conditioning
*Moringa oleifera* Seedcake Extract	The extract of the seedcake of *M. oleifera*	• skin conditioning—emollient
		• humectant. skin conditioning
*Saccharomyces*/*Moringa oleifera* Seed Oil/Glycerin Ferment Filtrate	The filtrate of the product obtained by the fermentation of *M. oleifera* seed oil and glycerin by the microorganism. *Saccharomyces*	• skin conditioning
		• skin conditioning - emollient
Hydrogenated Moringa Glucoside	The end product of the controlled hydrogenation of the product obtained by the condensation of Glucose with the fatty alcohols derived from *M. oleifera* seed oil	• cleansing
		• surfactant—emulsifying
		• surfactant—cleansing
(Broccoli/Cabbage/*Eruca sativa*/Radish) Sprout/*Moringa oleifera* Leaf Extract	The extract of the sprouts of *Brassica oleracea italica*. *Brassica oleracea capitata*. *Eruca sativa* and *Raphanus sativa*; and the leaves of *M. oleifera*	• antioxidant
[*Lactobacillus*/(*Cirsium japonicum*/*Moringa oleifera* Leaf) Extract Ferment and Sucrose] Extract Filtrate	The filtrate of the extract of a mixture of sucrose and the product obtained by the fermentation of an extract of the whole plant of *Cirsium japonicum* and the leaves of *M. oleifera* by the microorganism *Lactobacillus*	• antioxidant
		• skin conditioning—emollient
		• skin conditioning

## 10 Application in the food industry

Due to the high content of nutrients, *M. oleifera* is described in many sources as an alternative food source, particularly in the fight against malnutrition in developing countries (Ijarotimi et al., 2013; Karim et al., 2015). Energy bars and oil cake containing *M. olifera* are also sold in India (Ijarotimi et al., 2013; Karim et al., 2015).

*M. oleifera* can be used for food fortification. It is about adding vitamins, minerals and other essential nutrients to food products for enrichment. Such an enriched product should still have consistent sensory properties (Allen et al., 2006). Kalowole et al. described the enrichment of wheat dough with a maximum of 8% *M. oleifera* leaf powder in order to maintain the sensory qualities of the dough. A more promising alternative is the use of seeds or flowers, as the leaf powder is too intensely colored (Kolawole et al., 2013; Karim et al., 2015).

“Amala” cake is a staple of nutrition in many parts of Africa. The use of powdered *M. oleifera* leaves in this dough at a concentration of 10% increased the protein content by about 48%. The content of calcium, potassium, magnesium, sodium and iron also increased. Unfortunately, the addition of *M. oleifera* affects the sensory attributes of the dough (Awoyale et al., 2010; Ramota and Ojo, 2013; Karim et al., 2015).

Cereal gruel with the addition of *M. oleifera* is used as a complementary food for infants and for the production of breakfast cereals. It was found that the addition of powdered leaves of *M. oleifera* can improve the nutritional value of the gruel. It increased the content of vitamin A and protein, iron, calcium and phosphorus. Importantly, the addition of 10% powdered leaves *M. oleifera* did not significantly change the sensory qualities of this food (Olorode and Idowu, 2013; Arise et al., 2014; Abioye and Mo, 2015).

Including powder from flowers, seeds or leaves of *M. oleifera* in bread recipes allows to make bread on its own or in combination with other flours. Moreover, the addition of *M. oleifera* increases the amount of protein and crude fiber. The only problem associated with the production of bread with *M. oleifera* are the changed sensory properties, changes in the color of the crust a decrease in the height of the loaf compared to breads made of wheat flour, or the herbal aroma of the bread. A taste-masking flavoring agent is used to change the herbal flavor (Chinma et al., 2014).

*M. oleifera* can also be used to increase the nutrient content in dairy products such as yogurt and cheese. The addition of 0.5% extract from *M. oleifera* leaves in yogurt is considered sensory acceptable. Another product that can be enriched by the addition of *M. oleifera* is cheese. The content of protein, carbohydrates and fat is increased when *M. oleifera* leaf powder is added to cheese made from buffalo milk. The authors of the research on this product suggest the addition of a maximum of 2% of *M. oleifera* leaves in order to maintain the sensory properties. It is worth emphasizing that some consumers would also accept a higher concentration due to the herbal flavor of the products (Salem et al., 2013; Hekmat et al., 2015; Lokapirnasari et al., 2021).

*M. oleifera* leaves have also found their application in the preparation of soups, teas and salads. However, this usage is not well understood and the studies on this type of application are still ongoing. For now, it is not possible to say exactly what sensory and nutritional values it would have (Stevens et al., 2013; Babayeju et al., 2014).

## 11 Biofuels

*M. oleifera* is one of the plants for which the oil is suitable for the production of biodiesel (Ofor and Nwufo, 2011). Jatropha and Biodiesel Promotion Center (CJP, India) (Jatropha Biodiesel, 2023) has developed a specific mass propagation technique for *M. oleifera* for the production of biodiesel by micro-cutting. This company is constantly striving to improve practices that enable high yields of oil. CJP scientists are working to perfect agronomy, horticulture and plant genetics to introduce new highly productive varieties. In 2021, the Advanced Biofuel Center (ABC), also referred to as Moringa India, released a brochure “Strategies for the Commercialization of Moringa”. It contains information on husbandry practices that are used to maintain efficient and effective production. It develops various cultivation technologies and cultivation techniques for: harvesting leaves, harvesting seeds for oil, and harvesting both leaves and seeds of the plant (Jatropha World, 2023).

The Center for Advanced Biofuels, based on the conducted scientific research, selected a high-yielding perennial variety (cultivar) of *M. oleifera*—cv. MOMAX3. This variety provides the highest yield in seed and oil cultivation. *M. oleifera* cv. MOMAX3 are about three times more productive in seeds compared to their average harvested from *M. oleifera*. This is possible due to the large number of seeds in one fruit, their relatively large mass (over 30 g in 100 seeds), early flowering and rapid germination of the plant. The annual seed production per hectare is 2–3 tons, but can be as high as 8 tons, whereas *M. oleifera* normally produces about a ton of seeds per hectare per year. It is worth noting that the seeds are not genetically modified. They have been developed by the scientific method of selection (Jatropha World, 2023). The Center for Advanced Biofuels also selects an annual variety—*M. oleifera* cv. PKM1, which is less popular but very productive and recommended for the leaf production (Jatropha World, 2023).

In addition, other studies have been conducted in Australia to demonstrate the environmental benefits of growing *M. oleifera* for biodiesel production. This analysis was aimed at evaluating the environmental performance of the production of 1,000 L of biodiesel obtained from *M. oleifera* oil seeds. The study was carried out both in dry and irrigated areas. It was found that approximately 1 kg of *M. oleifera* seeds should be used to produce 0.33 L of seed oil. To obtain 1,000 L of biodiesel, 1,000 L of oilseed oil and 100 L of methanol are used. It was therefore calculated that approximately 3,030 kg of seeds should be used to obtain the amount of biodiesel needed for the research. Studies have shown that the greenhouse gas emissions from *M. oleifera* seed biodiesel production are 13% higher under irrigated conditions than under dry conditions. This is due to the use of greenhouse gas-producing agricultural machinery, which is used more frequently during irrigation. Biodiesel from *M. oleifera* oilseeds can greatly help in the fight against global warming (Amouri et al., 2023).

## 12 Safety of use

With the popularization of *M. oleifera* as an ingredient in food and cosmetics and its possible use in many diseases, the issue of its safety has arisen. Safety studies focus mainly on the use of *M. oleifera* as a dietary supplement. Most often, they are carried out on *M. oleifera* leaf extracts, but more research is still needed on extracts from individual parts of the plant and their safety. It is also worth noting that cosmetics in which *M. oleifera* is used must undergo appropriate safety tests for their use on the skin so that they can also enter the cosmetics market. Adedapo et al. conducted a safety study of an aqueous extract of *M. oleifera* leaves. The extract was tested for oral toxicity and for subacute toxicity on histological, hematological and biochemical parameters. The studies were conducted on rats. The acute toxicity experiment did not cause death in animals even at a dose of 2000 mg/kg. Oral toxicity was tested at doses of 400, 800 and 1,600 mg/kg. Significant changes in many parameters were noted: hemoglobin percentage, packed cell volume, mean blood cell hemoglobin concentration, mean red cell volume, total and differential WBC, liver enzyme levels, total protein levels, and bilirubin levels. In the study of platelet levels, the water extract from *M. oleifera* leaves did not cause significant changes. In addition, the body weight of the experimental animals also changed. There were no changes in significant organs. The results proved that *M. oleifera* leaf extract is relatively safe to use for nutritional and medicinal purposes (Adedapo et al., 2009).

Another investigation of the toxicity of an aqueous extract from the plant’s leaves was a study by Awodele et al. (2012). In the acute toxicity study, the extract was administered to mice orally at a dose of up to 1,640 mg/kg and intraperitoneally at a dose of up to 2000 mg/kg. A 60-day chronic toxicity study was also performed. Mice received the extract at doses of 250, 500 and 1,500 mg/kg. The lethal dose LD50 (causing death in 50% of the tested individuals) was estimated at 1,585 mg/kg. In all tested haematological parameters and biochemical tests, administration of the extract did not cause significant differences. The authors of the study concluded that the aqueous extract of *M. oleifera* leaves is safe (Awodele et al., 2012). Asiedu-Gyekye et al. measured the content of microelements and macroelements in dried leaves of *M. oleifera* and assessed its toxic effects on animals. Experimental rats were given 40–5,000 mg/kg of leaf extract to determine toxicity. Animal blood samples were evaluated for biochemical and hematolological parameters. In both acute and subacute toxicity studies, no adverse effects were observed. However, the authors concluded that due to the high content of elements, the intake of *M. oleifera* extract should not exceed 70 g per day to prevent the accumulation of elements that could cause toxicity (Asiedu-Gyekye et al., 2014). Ali et al. conducted a study on the skin irritating potential of *M. oleifera* leaf extract. *In vivo* evaluation of skin irritation by patch test and long-term evaluation of erythema was performed for a cream with 3% *M. oleifera* leaf extract. The test product was safe for topical application, and the cream had soothing effect on erythema (Ali et al., 2013). The evaluation of the genotoxicity of *M. oleifera* seed powder extract was performed by Rolim et al. (2011). The tests showed no signs of genotoxicity of the extract. However, the authors of the study emphasize. that when used in high concentrations, it may pose a health risk (Rolim et al., 2011). Ajibade et al. conducted a safety study of methanol extracts from *M. oleifera* seeds. The experiment was performed on rats to determine acute and subacute toxicity. In the acute toxicity study, adverse effects were observed at a dose of 4,000 mg/100 g b. w. (body weight) mortality was found after administration of 5,000 mg/100 g b. w. methanolic extract of *M. oleifera* seeds. For concentrations lower than 3,000 mg/100 g b. w. no side effects were observed. In a subacute toxicity study, a decrease in body weight was noted in animals after administration of 1,600 mg/100 g. The authors stated that *M. oleifera* seed extract is safe as a nutritional product and it is possible to use it in medicine (Ajibade et al., 2013). Studies by Luqman et al. (2012) on leaves and fruits *of M. oleifera* showed that the fruit in doses of up to 100 mg/kg of b.w. of *M. oleifera* fruit extract are safe and should show only beneficial properties (Luqman et al., 2012). Athikomkulchai et al. tested the safety of *M. oleifera* seed oil on the skin. The developed cream had anti-radical properties and did not cause any adverse reactions (Athikomkulchai et al., 2021).

In the EFSA documents (EFSA, 2023) the information about the usage of dried *M. oleifera* extract used in India as an abortifacient can be found. However, there is not much information about this effect in scientific publications. EFSA officially states that *M. oleifera* is recognized as safe (EFSA, 2015). The FDA, (2023) only provides information on “the dangers of *M. oleifera* supplements purchased from certain manufacturers.” As is well known, dietary supplements do not have the obligation of such restrictive tests as in the case of drugs. Hence the information that the consumer is misled by some manufacturers of supplements containing *M. oleifera*.

In conclusion, studies conducted on *M. oleifera* confirm its safety. However, this species is not as common as other plants used in supplements due to the lack of enough safety research on extracts from each part of the plant.

## 13 Biotechnological research

Currently, there is an increasing demand for plant species important from the therapeutic point of view. To this end, researchers are increasingly developing micropropagation protocols for plants with valuable properties. Proper shoot regeneration is essential to initiate clonal propagation and/or genetic transformation. Plant biotechnology allows for the cultivation of plants under constant conditions, e.g., temperature, lighting, which may affect the content of biologically active compounds. Biotechnological research conducted on *in vitro* cultures of *M. oleifera* is aimed at developing plant micropropagation protocols (Smetanska, 2008; Karuppusamy, 2009).

In a study by Marfori et al. mature green *M. oleifera* pods were used. In the preparation phase, the pods were thoroughly washed and their surface sterilized. Seeds were applied on Murashige and Skoog (MS) medium with 30 g sucrose and 5 g agar. Cultures were grown for 1–2 weeks and kept in the dark at 27°C. After germination, the seedlings were transferred and grown under white light. After a month, the sprouted seedlings were harvested and transferred to a sprout-inducing medium. MS sprout induction medium contained 6-benzlaminopurine (BAP) and thidiazuron (TDZ) or kinetin (Kin) at a concentration of 0–10 mM. Shoot and roots were transferred to the MS inducing medium containing 1-naphthaleneacetic acid (NAA), indolebutyric acid (IBA) or indoleacetic acid (IAA) at concentrations of 0–5 µM. Rooted cuttings were transferred to fresh induction medium for continuous shoot production or to soil. Studies have shown that BAP at a concentration of 2.5 µM induced the largest number of shoots (4.6 shoots per explant). It was greater than with TDZ and Kin. For Kin, the highest score was recorded using a concentration of 5 µM (2.4 shoots per explant). TDZ was most effective at a concentration of 2.5 µM (2 shoots per explant). In the next step, the seedlings induced the highest number of roots after applying 0.25 µM NAA (6.8 roots per shoot after 1 week). In the literature there are noticeable differences in the proposed plant growth and development regulators (PGRs) used for *M. oleifera* micropropagation processes (Marfori, 2010). According to Fahey and Stephenson the best growth of *M. oleifera* microcuttings occurs with 4.44 µM BAP and 2.68 µM NAA (Stephenson and Fahey, 2004). On the other hand, Islam et al. found that the best results were obtained when using 4.44–6.66 µM BAP for shoot induction. and their rooting is best in a PGR-free culture. Differences in the plant source and type of explants used may be responsible for the discrepancies (Islam et al., 2021).

In another study by Saini et al. worked on methods for rapid *in vitro* regeneration of *M. oleifera* and evaluated the yield of nutrient-enriched tissue cultures. The study was conducted on healthy, uniform *M. oleifera* seeds from India. These seeds were surface sterilized by immersing them in 0.1% Mercury chloride for 2 min and in 20% sodium hypochlorite for 10 min. The tested seeds were rinsed three times with distilled water, then the seed coats were removed and sterilized again for 5 min in 20% hypochlorite, then rinsed three times with distilled water. The sterilized seeds were placed on MS medium containing 30 g/L sucrose solidified with 5 g/L agar (pH was 5.8). The cultures were kept in the dark for 15 days at 27°C and then, after germination, were transferred to white fluorescent lamps. Shoots were induced by using sprouted cuttings of 3-4 nodes 3 or 4 weeks after inoculation. The explants were transferred to an MS induction medium containing tria-contanol at a concentration of 0–11.39 nmol and 0–8.88 nmol BAP and 0–5.37 μmol NAA to determine their effect on shoot formation. After 15 days, the number and length of shoots were assessed. Then, the obtained microshoots were repeatedly passaged on MS medium containing 4.44 nmol BAP. The next step was rooting the shoots. Segments with induced shoots were transferred to root-inducing MS medium containing IAA at a concentration of 0–5.71 nmol and optionally IBA at a concentration of 0–4.92 µmol. After 7 days, the number of roots per shoot and the length of roots were recorded. One-year-old *M. oleifera* rooted and transplanted *in vitro* was used to obtain field results. α-Tocopherol, carotenoids and chlorophyll were extracted. In the results of the study, the authors state that with the use of 4.44 μmol BAP, the largest number of shoots was induced (9 shoots per explant). Other substances used were less effective. The highest number of roots—15—was recorded after 7 days using IBA at a concentration of 4.92 nmol and IAA at a concentration of 2.85 nmol. In a plant nutrient study, it was determined that tissue culture increased the amount of nutrients from 5.5% to 14.7% compared to control culture. The high content of nutrients was due to the intensive vegetative growth of plants propagated *in vitro* (Saini et al., 2012).

*M. oleifera* is subjected to numerous scientific studies, but there is still a lack of research on the use of *in vitro* cultures in cosmetics. A study by Zanell et al*.* presents information on the induction of antioxidant metabolites in *M. oleifera* callus by abiotic stress. *M. oleifera* leaf callus was cultured *in vitro* and then various methods of inducing abiotic stress were tested. Exposure to high and low temperatures (4°C and 45°C) was applied, however, it did not cause any changes in terms of antioxidant metabolites. Then the callus was treated with salicylic acid at a concentration of 200 µmol and NaCl at a concentration of 50–100 µmol. The study lasted 30 days. The highest production of metabolites in callus from *M. oleifera*was found after 30 days of using NaCl at a concentration of 100 μmol, this was equal to the dose measured in a fresh *M. oleifera* leaf. High concentrations of NaCl (200 µmol) were able to inhibit callus growth and reduce the amount and bioactivity of its secondary metabolites (Zanella et al., 2019).

Mathur et al. conducted a study on callus cultures *in vitro M. oleifera*. Cultures were initiated from *M. oleifera* seeds and nodal segments on MS medium. Various concentrations and combinations of auxins and cytokinins were used. The best callus induction was found with 3 mg BAP and 3 mg IBA. Callus rooting in terms of morphogenesis was observed in MS media with 2 mg/L BAP and 0.2 mg/L KIN and 3 mg/L IBA and 0.5 mg/L NAA. Rooted plants were transferred to greenhouse conditions, after transplanting the seedlings, as much as 95% of the seedlings survived (Mathur et al., 2014).

## 14 Potential application in agriculture

Water extracts from the leaves, roots and pods were tested against 7 phytopathogenic fungi: *Fusarium oxysporum, Fusarium solani, A. solani, A. alternata, Rhizoctionia solani, Sclerotium rolfsii* and *Macrophomina phaseolina*. An agar medium test was used for the study. *M. oleifera* root extracts were used at concentrations of 5, 10, 15, 20% and 25%. Leaf and pod extracts were used at concentrations of 10, 20, 30, 40% and 50%. On 6 mm agar discs with hyphae of each of the above mushrooms, 20 mL of extracts were added. The percent inhibition of mycelial growth was calculated. In addition, a test was performed using the dry weight of the mycelium. It consisted in mixing an aqueous extract of roots, leaves and pods with sterilized potato broth medium, obtaining concentrations of: 0.5%; 1%; 1.5%; 2%; 2.5%; 5%; 10%; 15%; 20%, and 25%. Oil and *M. oleifera* seed extract were dosed in portions from 50 to 250 mL in an Erlenmeyer flask. The agar disk with fungal hyphae was transferred to flasks with *M. oleifera* extracts and incubated. The pathogen reduction effect was different and depended on the concentration of extracts containing *M. oleifera*. Higher antifungal activity was obtained using higher concentrations of extracts, but in the case of testing with dry weight, the results were the most promising and reduced the mycelium by up to half. The results indicate that *M. oleifera* can be used as a natural fungicide, a powerful biofungicide. The minimum inhibitory concentration (MIC) for the root was 25% and was 100% inhibitory on *F. oxysporum*, *F. solani, A. solani. A. alternata*, more than 90% *R. solani*, *S. rolfsii* and 87.4% *M. phaseolina*. For the leaf and pod extract, the MIC was 50% and had an inhibitory effect of 100% on *F. oxysporum*, *F. solani*, over 90% on *A. solani* and over 80% on *A. alternata*, *R. solani*, *S. rolfsii*, *M. phaseolina* (El-Mohamedy and Abdalla, 2014).

The latest study aimed to determine the antifungal potential of *M. oleifera* against *Botrytis cinerea*. Methanol and ethanol extracts from dried leaves and seeds were tested. The extracts were plated with five-day-old cultures of *B. cinerea* mycelia on agar plates (100 mg/mL). The plates were incubated at 28°C for 5–7 days. The MIC was determined by the Broekaert method using a microdilution technique or a microtiter plate. Studies have shown a highly inhibitory effect (99%) on the growth of *B. cinerea* when using an ethanol extract of the leaves. The MIC was 5 mg/mL and inhibited 100% of the fungal embryos. The minimum fungicidal concentration (MFC) of the ethanol extract from the leaves was 10 mg/mL (Ahmadu et al., 2021).

*M. oleifera* seeds were tested for their antibacterial activity against the *S. aureus* strain. The potential effect of seeds as a magnetic coagulant in the elimination of *S. aureus* in the treatment of dairy wastewater was assessed. 4,990 mL of distilled water and 10 mL of UHT milk were placed in a sterile container. The solution was homogenized by hand and then 5 µL of active *S. aureus* culture was added. The following evaluations were made: pH, temperature, color, spectrophotometric evaluation of turbidity, microbiological load test. Iron sulfate and iron nitrate nanohydrate were homogenized in distilled water, then sodium hydroxide was added. ${\text{Fe}}_{3}O_{4}$ nanoparticles were created. A saline extract of *M. oleifera* seeds was obtained by crushing, grinding and homogenizing the seeds with sodium chloride solution, which was then filtered. To obtain *M. oleifera* and ${\text{Fe}}_{3}O_{4}$ magnetic coagulant, the extracts were mixed for an hour at room temperature. Sixteen tests were carried out using the composition of ${\text{Fe}}_{3}O_{4}$ and *M. oleifera* added to dairy wastewater in various concentrations. Color and turbidity were assessed spectrophotometric examination was performed at *λ* = 254 nm. The growth of *S. aureus* was assessed on the sewage surface after 30 min of sedimentation. The results of the study confirmed the effective elimination of *S. aureus* from the surface of sewage and indicate that *M. oleifera* together with ${\text{Fe}}_{3}O_{4}$ can be used as a natural coagulant due to its ability to remove bacteria (Oliveira et al., 2021).

## 15 Summary

*M. oleifera* is a species of a tropical tree with an increasing importance in the food, pharmaceutical, cosmetic, and agricultural industries. Thanks to its outstanding health-promoting properties, it has earned a few nicknames, like ‘the miraculous tree’ and ‘the tree of long life’. The raw material most often obtained from *M. oleifera* is the leaf, however, other parts have been used as raw materials, including its fruit, flower, bark, seed and root.

*M. oleifera* provides raw materials with high nutritional value. *M. oleifera* seed oil used in addition to the food industry in the cosmetic industry due to its conditioning properties. The chemical profile is dominated by: proteins (amino acids such as arginine and serine), fatty acids (omega-3 and omega-6), vitamins (vitamin A, B and C and tocopherols), minerals (calcium, magnesium, sodium, potassium) and valuable polyphenolic compounds like phenolic acids (gallic acid, ferulic acid) and flavonoids (myricetin, rutoside, kaempferol).

The latest scientific research confirms the health-promoting properties of *M. oleifera* known from traditional medicine of India, China and African countries, such as: antioxidant, antibacterial, anti-inflammatory, hepatoprotective and antidepressant effects. In addition, research indicates other valuable properties of the raw material, such as neuroprotective, anti-diabetic, accelerating wound healing, anti-psoriatic or anti-cancer properties. *M. oleifera* as a food plant has descriptions in FDA and EFSA documents. The safety data indicate that *M. oleifera* is used in health, food and cosmetic products with high confidence.

The growing interest of the food and cosmetics industry as well as numerous studies proving the valuable biological properties of *M. oleifera* may result in greater interest in this species in the medicinal industry in the future.
