# Supplementary material for: Moringa oleifera (drumstick tree)—nutraceutical, cosmetological and medicinal importance: a review
Source: Front Pharmacol. 2024 Feb 2;15:1288382. doi: 10.3389/fphar.2024.1288382 (PMC10869624; doi:10.3389/fphar.2024.1288382)
Supplement: Supplementary file 1 [file DataSheet1.pdf]

Supplementary Material

Moringa oleifera Lam. (drumstick tree) – the complex overview on nutraceutical, cosmetological and medicinal importance – a review

Marta Klimek-Szczykutowicz[1], Katarzyna Gaweł-Bęben[2], Angelika Rutka[3], Eliza Blicharska[4], Małgorzata Tatarczak-Michalewska[4], Katarzyna Kulik-Siarek[1], Wirginia Kukula-Koch [5], Magdalena Anna Malinowska[6], Agnieszka Szopa[3*]

[1] Department of Dermatology, Cosmetology and Aesthetic Surgery, The Institute of Medical Sciences, Medical College, Jan Kochanowski University, IX Wieków Kielc 19a, 25-516 Kielce, Poland

[2] Department of Cosmetology, University of Information Technology and Management in Rzeszów, Sucharskiego 2, 35-225 Rzeszów, Poland

[3] Chair and Department of Pharmaceutical Botany, Faculty of Pharmacy, Jagiellonian University, Medical College, Medyczna 9, 30-688 Cracow, Poland

[4] Department of Pathobiochemistry and Interdisciplinary Applications of Ion Chromatography, Biomedical Sciences, Medical University of Lublin, 1 Chodźki Str., 20-093 Lublin, Poland

[5] Department of Pharmacognosy with Medicinal Plants Garden, Medical University of Lublin, 1 Chodzki str., 20-093 Lublin, Poland

[6] Institute of Organic Chemistry and Technology, Faculty of Chemical Engineering and Technology, Cracow University of Technology, 24 Warszawska St., 31-155 Cracow, Poland
*** Correspondence:**
Agnieszka Szopa
a.szopa@uj.edu.pl

1. List of *Moringa oleifera* names in different languages

M. oleifera has four accepted and commonly used synonymous names in Latin (Ramachandran et al., 1980; World Flora Online, 2022):

- *Moringa zeylanica* Burmann,
- *Hyperanthera moringa* (L.) Vahl,
- *Guilandina moringa* L.,
- *Moringa pterygosperma Gaertner.*
-

Nomenclature of *M. oleifera* in traditional medicine:

- Traditional Chinese Medicine: *„La ken”,*
- Unani: *„Sahajan”,*
- Ayurveda: *„Haritashaaka”, „Raktaka”, „Akshiva”.*

Traditional regional names, the origin of which is missing (Ramachandran et al., 1980; Sujatha and Patel, 2017):

- *Moonga,*
- *Mulangay,*
- *Nebeday,*
- *Shigon.*

M. oleifera in Polish is called:

- *moringa olejodajna,*
- *drzewo chrzanowe.*

Names are used in English (Ramachandran et al., 1980):

- *Benzolive tree,*
- *Drumstick tree,*
- *Horseradish tree,*
- *Maranga calalu,*
- *Mother's Best Friend,*
- *Never die tree,*
- *Radish tree,*
- *West Indian Ben tree.*

Names in German (Trees for Life):

- *Behenbaum,*
- *Ben boom,*
- *Flügelsaniger,*
- *Bahenussbaum,*
- *Bannessbaum,*
- *Peperwortel boom,*
- *Pferderettichbaum.*

Names in French (Fuglie, 1999):

- *Arbre radis du cheval,*
- *Bambou-bananier,*
- *Bèn ailé,*
- *Benzolive,*
- *Benzolivier*
- *Ben oléifère,*
- *Doliv,*
- *Graines benne,*
- *Maloko,*
- *Moloko,*
- *Morungue,*
- *Moringe a graine ailee,*
- *Olivier.*

Chinese names (Fuglie, 1999):

- *„Ia mu”,*
- *„La ken”.*

Names in Spanish (also used in Latin America) (Fuglie, 1999):

- *Acacia,*
- *Aceite,*
- *Aceite de Ben,*
- *Aceitoso,*
- *Angela,*
- *Arbol de los aspáragos,*
- *Árbol del ben,*
- *Arbol e las perlas,*
- *Azucarillo ,*
- *Bamboubamamoer,*
- *Ben,*
- *Brenolli,*
- *Calicita,*
- *Cedro,*
- *Cenauro,*
- *Chinto borrego,*
- *Chinto borrgo,*
- *Chuva de prata,*
- *Colirio,*
- *Desengaño,*
- *Flor de Jacinto,*
- *Gailito,*
- *Goma,*
- *Guaieña,*
- *Hoja de sen,*

- *Jacinto,*
- *Jeringa,*
- *La libertad,*
- *Leno nefrítico,*
- *Libertad,*
- *Maranga calalu,*
- *Marango,*
- *Maranjo,*
- *Marangon,*
- *Marenque,*
- *Mascar,*
- *Mawonga,*
- *Morenga,*
- *Moriengo,*
- *Moringa.*
- *Narango,*
- *Noz de bem,*
- *Orengo,*
- *Orselli,*
- *Palo blanco,*
- *Palo de geringa,*
- *Palo jeringa,*
- *Palo de abejas,*
- *Palo de aceite,*
- *Palo de Jeringa,*
- *Palo de Tambor,*
- *Paraíso blanco,*
- *Paraíso de Espana,*
- *Paraíso extranjero,*
- *Paraíso francés,*
- *Perla,*
- *Perlas,*
- *Perla de la India ,*
- *Perlas del oriente,*
- *Pois quinique,*
- *Quiabo de tres quinas,*
- *Sainto John,*
- *Recedad,*
- *Salaster,*
- *Salibau,*
- *San Jacinto,*
- *Seringa,*
- *Sen de la tierra,*
- *Sen.*

Names in Portuguese (Fuglie, 1999):

- *Acácia branca,*
- *Cedra,*
- *Marungo,*
- *Moringa,*
- *Moringueiro,*
- *Muringa.*
    -

Names in Italian (Fuglie, 1999):

- *Sàndalo ceruleo*

Names in Indonesian, depending on the region (Fuglie, 1999):

- *Baoe fo,*
- *Kafok,*
- *Kalor,*
- *Kai fok,*
- *Kawona,*
- *Kelo,*
- *Kelor,*
- *Kerore,*
- *Maroenga,*
- *Moltong,*
- *Motong,*
- *Oege Kelo,*
- *Oho Gaairi,*
- *Remoenggai,*
- *Sajor Kelor,*
- *Tjelor,*
- *Wona.*

In Hindi (India) (Fuglie, 1999):

- *Danshamula,*
- *Lal Sahinjano,*
- *Mangnai,*
- *Midho-saragavo,*
- *Moosing,*
- *Moringa,*
- *Morunga,*
- *Morungai,*
- *Morunna,*
- *Mosing,*
- *Mrongo*
- *Mulaka,*
- *Mulgia,*

- *Munaga,*
- *Munga ara,*
- *Munigha,*
- *Muringa,*
- *Murinna,*
- *Murungai,*
- *Murunkak-kai,*
- *Noogay,*
- *Nugga egipa,*
- *Nugge,*
- *Nuggekai,*
- *Nuggekodu,*
- *Nuggi Mara,*
- *Saihan,*
- *Sahija,*
- *Saragavo,*
- *Saragvo,*
- *Saijna,*
- *Sajina,*
- *Sajna,*
- *Sahajna,*
- *Sanjina,*
- *Sanjna,*
- *Sarinjna,*
- *Segra,*
- *Sejana,*
- *Shajmah,*
- *Shajna,*
- *Shevga,*
- *Shivga,*
- *Shobanjana,*
- *Sigru,*
- *Sigru Shobhanjan,*
- *Soandal,*
- *Soanjana,*
- *Sobhan jana,*
- *Sojna,*
- *Sujana,*
- *Sujna,*
- *Sunara,*
- *Sundan,*
- *Suragavo,*
- *Swanjera,*
- *Sweta Maricha.*

Names in the languages spoken in the Togolese Republic (Fuglie, 1999):

- *Amedoti,*
- *Bagaelean,*
- *Baganlua,*
- *Ekpoti,*
- *Gambadaduk,*
- *Jevoti,*
- *Jovoviti,*
- *Kpoti,*
- *Kpotima,*
- *Mágurua maser,*
- *Molo-Kpoti,*
- *Yevu-ti,*
- *Yovoviti.*

Names in languages spoken in Senegal (Fuglie, 1999):

- *Binêbeddai,*
- *Binêbeddaï,*
- *Nébèdayo,*
- *Nédèdayo,*
- *Némédayo,*
- *Névrédayo,*
- *Nébédaï,*
- *Nébédai,*
- *Nèbédaï,*
- *Nébéday,*
- *Névoidai,*
- *Névoïdaï,*
- *Nöbödaï,*
- *Nedôday,*
- *Neböday,*
- *Neverday,*
- *Sap-Sap.*

Names in languages spoken in Nigeria (Fuglie, 1999):

- *Adagba malero,*
- *Bagaruwar maka,*
- *Bagaruwar masar,*
- *Barambo,*
- *Chigban Wawa,*
- *Ewele,*
- *Ewé ilé,*
- *Ewe igbálé,*
- *Idagbo moneyé,*

- *Gawara,*
- *Gaware,*
- *Habiwal hausa,*
- *Konamarade,*
- *Koraukin zaila,*
- *Odudu oyibo,*
- *Oku-ghara-ite,*
- *Okochi egbu,*
- *Okwe oyibo,*
- *Okwe olu,*
- *Rimin nacara,*
- *Rini maka,*
- *Samarin danga,*
- *Shipka hali,*
- *Shuka halinka,*
- *Uhe,*
- *Zogalla,*
- *Zogalla-gandi.*

In the languages spoken in Niger (Fuglie, 1999):

- *Alim,*
- *Halim,*
- *Windi-bundu,*
- *Zôgala gandi,*

In languages spoken in various other African countries not listed above (Fuglie, 1999):

- Benin – *Guildandeni, Latj iri, Legi-lakili, Kotba, Yovo vigbe, Yovo Kpati,*
- Burkina Faso – *La – Banyu,*
- Chad – *Kag n'dongue,*
- Ethiopia – *Shalchada, Shelagda,*
- Ghana – *Gambadua, Nasadua, Kpokpotsor,*
- Cameroon – *Chabané, Paizalava,*
- Kenya – *Mborongi,*
- Malawi – *Chakate, Maula tengo, Mbula, Mpempu, Mpenba, Mpundi, Muula, Sangoa, Shangoa,*
- Mozambique - *Mvungué,*
- Comoros Islands – *Anambo, Mvungué.*

In less common languages, *M. oleifera* is named (Fuglie, 1999; Parrotta, 2009; Paikra et al., 2017):

- Abenaki language – *Kpashima,*
- Arabic language – *Alim, Halim, Moringa, Rawag, Saisam, Shagara al. Ruwag, Shagara zaki al moya,*
- Assamese language – *Saijna, Sohjna,*
- Amharic language – *Sheiferaw, Shiferaw,*
- Antillean language – *Boganja, Dangap, Drède mouroungue,*

- Bambara language – *Névrédé, Gnougou Jirini, Manjirini, Massa Jirini, Kandjirini,*
- Bariba language - Yuru ara, Yorwata, Yoroguma,
- Bengali language – *Sajina,*
- Burmese language – *Daintha, Dandalonbin, Dandalun – bin, Dandalun,*
- Chamorro language – *Katdes, Malaungay, Malaungkai, Marronggai, Marungai, Marunggai,*
- Chewa language – *Cham'mwanba, Kangaluni, Chamwamba,*
- Dari language – *Obnukuo, Ornyyukuo, Zangala,*
- Dendi language – *Windibundu,*
- Dinka language – *Anid,*
- Diula language – *Ardjeneyiri, Ardjian jirri,*
- Dravidian language – *Morunga,*
- Ewe language – *Atiuwuse, Babati, Babatsi, Kpokpoti, Kpotowuzie, Yevu-ti, Yevutsi,*
- Fijian language – *Sajina*,
- Phone language – *Patima, Kpatima, Yovokpatin, Kpano, Yovotin,*
- Ful language – *Aljannahi, Guiligandja, Gigandjah, Gligandjahi,*
- Ginuch language – *Aleko, Haleko,*
- Gourmandche language -*Alj an-tiiga, Ki gambaga, Makkakomboanga,*
- Gujarachi language – *Suragavo,*
- Gün language – *Ékwè kpatin, Kpajima,*
- Hausa language – *Zingaridende, Zogalagandi,*
- Indo-Creole language – *Mouroungue,*
- Khmer language – *Daem mrum, Mrum, Mrom,*
- Creole language – Drède mouroungue,
- Konkani language – *Maissang, Moring, Moxing,*
- Lomwe language – *Sangoa, Shangoa,*
- Mafa language – *Gagawandalahai,*
- Malay language – *Murinna*, *Sigru,*
- Malagasy language – *Anamambo, Anamorongo, Feliimorongo, Felikambo, Felikamoranga, Landihazo, Moringa, Moringy,*
- Mandarin language– *Djhiré,*
- Marathi language – *Achajhada, Shevgi,*
- język mossi – *Argentiga, Alsam tiga* (rajskie drzewo) *, Arzan tiiga, Alsan-tügam arzantiga,*
- Moundang language –*Naa-toukoré,*
- Naten language – *Tekpinda,*
- Nepali language – *Shobhanjan, Sigru,*
- Palau language – *Malungkai,*
- Punjabi language – *Sainjna, Soanjna,*
- Sankrit language – *Subhajana, Sigru,*
- Segou language – *Verdaye,*
- Swahili language – *Mboga chungu, Mijungu moto, Mlonge, Mlongo, Mkimbo, Mronge, Mrongo, Mzunze, Mzunzu, Shingo,*
- Sinhala language – *Murunga,*
- Thai language – *Kaanaeng-doeng, Phak eehuem, Phak eehum, Pak-nuea-kai, Se-cho-ya, Ma rum,*
- Tamil language – *Mulaga, Munaga, Murungai,*

- Telugu language – *Mulaga, Munaga, Tellamunaga,*
- Tonga language – *Mupulanga, Zakalanda, Zagalanda,*
- Toupouri language – *Naa-nko,*
- Urdu language – *Sahajna,*
- Waama language – *Yori ku-ounun fa,*
- Yao language – *Kalokola,*
- Yoruba language – *Agun, Ayere, Ewé igablé, Ewé ilé, Ewé oyibo, Manyieninu, Oyibo*
